# Law of large numbers for the drift of the two-dimensional wreath product

**DOI:** 10.1007/s00440-021-01098-6

**Published:** 2021-12-06

**Authors:** Anna Erschler, Tianyi Zheng

**Affiliations:** 1 Département de mathématiques et applications, École normale supérieure, CNRS, PSL Research University, 45 rue d’Ulm, 75005 Paris, France; 2 Department of Mathematics, UC San Diego, 9500 Giman Dr., La Jolla, CA 92093 USA

**Keywords:** 60F15, 60B15, 20F69

## Abstract

We prove the law of large numbers for the drift of random walks on the two-dimensional lamplighter group, under the assumption that the random walk has finite $(2+\epsilon )$-moment. This result is in contrast with classical examples of abelian groups, where the displacement after *n* steps, normalised by its mean, does not concentrate, and the limiting distribution of the normalised *n*-step displacement admits a density whose support is $[0,\infty )$. We study further examples of groups, some with random walks satisfying LLN for drift and other examples where such concentration phenomenon does not hold, and study relation of this property with asymptotic geometry of groups.

## Introduction

In this paper we study the limiting behavior of the distance to the origin of random walks on non-abelian solvable groups, in particular show a law of large numbers type result for random walks of sublinear drift on wreath products over ${\mathbb {Z}}^{2}$ with finite or infinite lamp groups. This is in contrast with classical central limit theorems for random walks on ${\mathbb {Z}}^{d}$, see e.g., [24, Chapter VIII.4].

Let *G* be a finitely generated group and *S* be a finite generating set of *G*. Denote by $l_{S}$ the word length function on *G* with respect to the generating set *S*. Let $\mu$ be a probability measure on *G*; consider a random walk $\left( X_{n}\right) _{n=0}^{\infty }$ on *G* with step distribution $\mu$. The *drift function* $L_{\mu }(n)$ is defined as the mean of $l_{S}(X_{n}),$ that is,

$$\begin{aligned} L_{\mu }(n)={\mathbb {E}}\left[ l_{S}(X_{n})\right] . \end{aligned}$$

If $L_{\mu }(n)$ grows linearly, then it is well-known that by Kingman’s subadditive ergodic theorem $l_{S}(X_{n})/n$ tends to a positive constant almost surely. If $L_{\mu }(n)$ is sublinear, then in general the sequence of normalized random variables $l_{S}(X_{n})/L_{\mu }(n)$ does not necessarily converge in distribution. See Proposition 6.6 for examples of finitely generated groups, on which a simple random walk $(X_n)_{n=0}^{\infty }$ satisfies that $l_{S}(X_{n})/L_{\mu }(n)$ converges in distribution to a limiting density with support $(0,\infty )$ along one subsequence $(n_i)$; and converges in probability to the constant 1 along another subsequence $(m_i)$.

When $L_{\mu }(n)\simeq \sqrt{n},$ we say the $\mu$-random walk is diffusive. In contrast to abelian groups, where symmetric simple random walks have diffusive behavior, there is a rich spectrum of behavior of $L_{\mu }(n)$ for symmetric random walks on non-abelian groups: for any subadditive function *f* between $\sqrt{n}$ and *n* satisfying certain regularity conditions, there exists a group *G* and a symmetric measure $\mu$ on *G* with finite generating support such that $L_{\mu }(n)$ is equivalent to *f*, see [2, 10].

In many examples, the limiting distribution of $l_{S}(X_{n})/L_{\mu }(n)$ exists and admits a density on ${\mathbb {R}}_{+}$. For example, this is the case when *G* is abelian and $\mu$ is a centered measure with finite generating support. This fact can be deduced from the local central limit theorem for random walks on ${\mathbb {Z}}^{d}$, see e.g. [34, Chapter 2.1].

When $l_{S}(X_{n})/L_{\mu }(n)$ converges to a constant $c>0$ almost surely, we say that the distance to the origin $l_{S}(X_{n})$ of the random walk obeys a law of large numbers. To our knowledge, it has not been studied up to now when such almost sure convergence of $l_{S}(X_{n})/L_{\mu }(n)$ to a constant occurs for random walks with sublinear drift function $L_{\mu }(n)$. In this paper, we show a law of large numbers for the displacement of a centered random walk on the wreath product ${{\mathbb {Z}}}^{2}\wr ({\mathbb {Z}}/2{\mathbb {Z}})$. Recall that the wreath product $H\wr L$ of groups *H* and *L* is a semi-direct product $\left( \oplus _{H}L\right) \rtimes H$, where *H* acts by translation on $\oplus _{H}L$. The group $H\wr ({\mathbb {Z}}/2{\mathbb {Z}})$ is sometimes called the lamplighter group over *H* (with the lamp group ${\mathbb {Z}}/2{\mathbb {Z}}$). We will recall this well-known interpretation as lamplighter in “plan of the proof” section at the end of this introduction.

The study of random walks on wreath products is initiated by Kaimanovich and Vershik [31], who have shown that wreath products illustrate several phenomena about Poisson boundary on random walks on groups. One and two dimensional wreath products ${\mathbb {Z}}\wr {\mathbb {Z}}/2{\mathbb {Z}}$ and ${\mathbb {Z}}^{2}\wr {\mathbb {Z}}/2{\mathbb {Z}}$ are examples of groups of exponential growth where the Poisson boundary of simple random walks is trivial; ${\mathbb {Z}}^{d}\wr {\mathbb {Z}}/2{\mathbb {Z}}$, $d\ge 3$, are amenable groups with non-trivial boundary of simple random walks; $G={\mathbb {Z}}\wr {\mathbb {Z}}/2{\mathbb {Z}}$ admits (infinite entropy) measures $\mu$ such that the boundary of $(G,\mu )$ is non-trivial and the boundary defined by an inverse measure $(G,{\hat{\mu }})$ is trivial. The study of Poisson boundary on wreath products was continued in a series of works, we mention that a complete description of the Poisson boundary of simple random walks on wreath product ${\mathbb {Z}}^d\wr {\mathbb {Z}}/2{\mathbb {Z}}$ is given in the work of the first named author [21] for $d\ge 5$; and more recently in Lyons and Peres [37] for $d\ge 3$. Many other questions about random walks on wreath products were studied. Here is a non-exhaustive list of problems: return probability on wreath products was studied in papers of Varopoulos [51], Pittet and Saloff-Coste [47] and Revelle [48], Law of iterated logarithm was studied in [49], positive harmonic functions and Martin boundary by Brofferio and Woess [11, 12], minimal growth of (not necessary bounded and not necessary positive) harmonic function by Benjamini et al. [5], recurrent subsets (and instability of recurrence of subsets and Green kernel) by Benjamini and Revelle [6]. Our main interest here are infinite wreath products, but we mention that random walks on finite wreath products also provide many interesting examples for the study of random walks on finite graphs, see for instance Häggström and Jonasson [28], Peres and Revelle [44] and Komjáthy, Peres [32] for the study of mixing and relaxation time for random walks on these groups; and Miller and Sousi [39], Dembo et al. [18] for the study of late points of random walks. In papers above and in our work, we study Markov kernels invariant by group actions. The work of Lyons et al. [36] about “ homesick” random walks on these groups do not satisfy this condition: a somehow counterintuitive example when inward based random walks move quicker from the origin than simple random walks. Finally, we always discuss here wreath products for the action of the group on itself and we do not mention several recent works about random walks on permutational extensions.

We say that a probability measure $\mu$ on a group *G* is *centered* if $\mu$ has finite first moment and $\sum _{g\in G}\chi (g)\mu (g)=0$ for any homomorphism $\chi :G\rightarrow ({\mathbb {R}},+)$. We say that a measure $\mu$ on *G* is non-degenerate if its support generates *G* as a semigroup. The measure $\mu$ has *finite* $\alpha$*-moment* if $\sum _{g\in G}l_{S}(g)^{\alpha }\mu (g)<\infty$. It is clear that this condition does not depend on the choice of the generating set *S*. Now we formulate our main result for the displacement of random walk on two-dimensional lamplighter groups.

### Theorem 1.1

Let $G={{\mathbb {Z}}}^{2}\wr ({\mathbb {Z}}/2{\mathbb {Z}})$ and $\mu$ be a centered non-degenerate probability measure of finite $(2+\epsilon )$-moment on *G*, $\epsilon >0$. Let *S* be a finite generating set of *G*. Then the $\mu$-random walk $(X_{n})_{n=0}^{\infty }$ on *G* satisfies

$$\begin{aligned} \lim _{n\rightarrow \infty }\frac{l_{S}(X_{n})}{n/\log n}=c \text{ a.s. } \end{aligned}$$

for some positive constant *c*.

Note that among the lamplighter groups ${{\mathbb {Z}}}^{d}\wr ({\mathbb {Z}}/2{\mathbb {Z}})$, the two-dimensional case is the only one to be considered in Theorem 1.1. Indeed when $d=1$, a symmetric nondegenerate random walk with finite second moment on ${{\mathbb {Z}}}\wr ({\mathbb {Z}}/2{\mathbb {Z}})$ has diffusive behavior, and moreover $l_S(X_n)/\sqrt{n}$ converges in distribution to a limiting density on $(0,\infty )$, see more discussion below. When $d\ge 3$, since the Poisson boundary is nontrivial by the result of Kaimanovich and Vershik [31] cited earlier, we have that $L_\mu (n)$ grows linearly and $l_S(X_n)/L_\mu (n)$ converges almost surely to a constant by Kingman’s subadditive ergodic theorem. We also remark that in Theorem 1.1 without the second moment condition the normalization can be different from $n/\log n$. For instance, there are symmetric random walks with finite first moment on $\mathbb {{\mathbb {Z}}}^{2}\wr ({\mathbb {Z}}/2{\mathbb {Z}})$ which have linear speed, see more explanation in Remark 4.1.

We also prove that the law of large numbers for random walk displacement holds in some examples with infinite lamp groups, for instance on the wreath product ${\mathbb {Z}}^{2}\wr {\mathbb {Z}}$. In that case the argument relies on a result of Černý [13], see Proposition 5.2. Our main goal is to treat the case where the lamp group is finite as in Theorem 1.1.

Now we comment on the situation in contrast with the phenomenon described in Theorem 1.1. By Gromov’s polynomial growth theorem [26], groups of polynomial growth are virtually nilpotent. In Alexopoulos [1], a local central limit theorem is established for centered finite range random walks on groups of polynomial growth. As a consequence, for centered finite range random walks on polynomial growth groups, $l_{S}(X_{n})/\sqrt{n}$ converges in distribution and the limiting distribution admits a positive density on the ray $(0,\infty ).$ Beyond groups of polynomial growth, such convergence in distribution can also be observed in some examples of groups of exponential growth. In Proposition 6.2 we compute the limiting distribution of $l_{S}(X_{n})/\sqrt{n}$ for symmetric random walks of finite second moment on ${\mathbb {Z}}\wr ({\mathbb {Z}}/2{\mathbb {Z}})$. Note that in the examples mentioned above, the random walks exhibit diffusive behavior (for an example with non-diffusive drift function where the limiting distribution of $l_{S}(X_{n})/L_{\mu }(n)$ can be computed see Example 5.1), it is natural to ask in general the following question.

### Question 1.2

Suppose the random walk $(X_n)_{n=0}^{\infty }$ on *G* is diffusive, that is $L_{\mu }(n)\simeq \sqrt{n}$. Is it true that $l_{S}(X_{n})/L_{\mu }(n)$ converges in distribution to a limiting law whose density charges the whole ray $(0,\infty )$?

It is known that the rate of escape of random walks on infinite groups always satisfy a diffusive lower bound, see [35]. In Sect. 6 we formulate and discuss this question under the stronger assumption that the group *G* admits what is called controlled Følner pairs (see Question 6.1). We provide some evidence supporting a positive answer. As mentioned earlier, Proposition 6.2 describes the limiting distribution of $l_{S}(X_{n})/L_{\mu }(n)$ for simple random walks on the lamplighter ${\mathbb {Z}}\wr F$ over ${\mathbb {Z}}$ with finite lamp group *F*. As another evidence, in Lemma 6.4, we show that if *G* admits a sequence of controlled Følner pairs and limiting density of $l_{S}(X_{n})/L_{\mu }(n)$ exists, then the support of the limiting density must be the whole ray $(0,\infty ).$ The definition of controlled Følner pairs is recalled in Sect. 6. If instead of a sequence of controlled Følner pairs, we assume a weakened condition that *G* admits controlled Følner pairs on some scales, then the situation is more complicated. We illustrate this by examples of lacunary hyperbolic groups which admit controlled Følner pairs on some scales, where the limiting behavior of $l_{S}(X_{n_{i}})/L_{\mu }(n_{i})$ depends on the choice of $(n_{i})$, see Proposition 6.6.

We mention that in certain classes of groups where $L_{\mu }(n)$ grows linearly, central limit theorems for $l_{S}(X_{n})-L_{\mu }(n)$ are established: see for example the central limit theorem by Benoist and Quint on hyperbolic groups [7]; and more generally on acylindrically hyperbolic groups by a different approach in Mathieu and Sisto [38]. Prior to these works, central limit theorem for the drift of random walk in the Green metric on a hyperbolic group is established by Bjorklund in [8], and for quasimorphisms along random walk trajectories in Bjorklund and Hartnick [9]. In Question 1.2 one might also ask (similar to the situation of abelian groups where the classical central limit theorem holds), whether the limiting density has Gaussian decay at infinity. See more on this in Sect. 6 and in particular a list of questions after Question 6.1.

### Plan of the proof of Theorem 1.1

For simple random walk on ${\mathbb {Z}}^{2}$, a classical result of Dvoretzky and Erdős [20] shows that the size of the range satisfies the strong law of large numbers:

1.1 $$\begin{aligned} \lim _{n\rightarrow \infty }\frac{R_{n}}{\pi n/\log n}=1 \text{ a.s., } \end{aligned}$$

where $R_{n}$ is the number of vertices visited by the random walk up to time *n*. This result is generalized in Jain and Pruitt [29] where it is shown that for any recurrent random walk on ${\mathbb {Z}}^{2}$, the strong law of large numbers $\lim _{n\rightarrow \infty }R_{n}/{\mathbb {E}}\left[ R_{n}\right] =1$ holds almost surely.

**Fig. 1:**
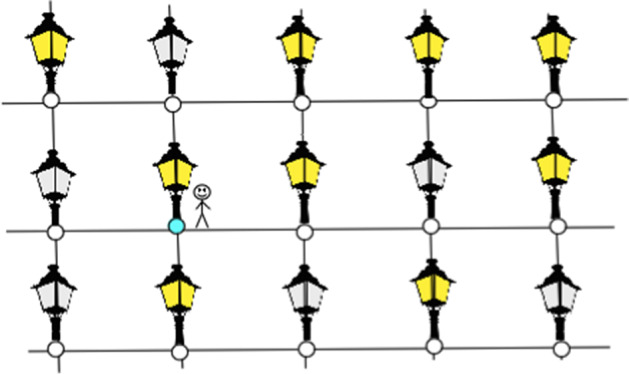
Vertices of the lamplighter group ${\mathbb {Z}}^2 \wr {\mathbb {Z}}/2{\mathbb {Z}}$ are pairs: a position of the walker in ${\mathbb {Z}}^2$ (here shown with a cyan circle) and a configuration of lamps, with finite number of lamps litten. The edges of the graph correspond either to steps of the walker in ${\mathbb {Z}}^2$ or to switching the lamp in the position of the walker (color figure online)

We recall that the name lamplighter for wreath product $H\wr {\mathbb {Z}}/2{\mathbb {Z}}$ with the two elements group ${\mathbb {Z}}/2{\mathbb {Z}}$ comes from the following observation. The elements of the wreath product are of the form (*h*, *f*), where $h\in H$ and $f:H\rightarrow {\mathbb {Z}}/2{\mathbb {Z}}$. One can view the function *f* as a configuration of “ lamps”, saying that in each element *h* of a base group *H* there is a lamp, which is on if the value *f*(*h*) is equal to 1 and off if this value is zero, see Fig. 1 This interpretation is in particular useful when we discuss simple random walks on $H\wr {\mathbb {Z}}/2{\mathbb {Z}}$: the random walker walks on *H*, and at the point he visits he can lit or extinguish the lamp, with some probability specified by the step distribution.

It is well known that the word length in a lamplighter group is closely related to the Travelling Salesman Problem (TSP) in the base group, see e.g. [43]. Therefore it is straightforward that the TSP arises in the study of the drift function of random walks. Basic estimates for the TSP can be useful to understand the behavior of random walks on lamplighter groups, in particular the asymptotics of the drift functions. We also mention that questions in metric geometry, in particular around distortions of embeddings of wreath products into Banach spaces, require deeper understanding of TSP. In Naor and Peres [40], the Jones traveling salesman theorem [30] is applied to construct embeddings of lamplighter groups into Banach spaces.

Denote an element *g* of the lamplighter group $G={\mathbb {Z}}^{2}\wr ({\mathbb {Z}}/2{\mathbb {Z}})$ by (*x*, *f*), where $x\in {\mathbb {Z}}^{2}$ and $f:{\mathbb {Z}}^{2}\rightarrow {\mathbb {Z}}/2{\mathbb {Z}}$ is a function of finite support which we refer to as the lamp configuration of *g*. Consider the standard generating set $S=\left\{ s_{1}=(e_{1},\mathbf{0}),s_{2}=(e_{2},\mathbf{0}),\delta =(0,\delta _{0}^{1})\right\}$, where $e_{1}=(1,0)$, $e_{2}=(0,1)$ form the standard basis of ${\mathbb {Z}}^{2}$, $\mathbf{0}$ is the constant function on ${\mathbb {Z}}^{2}$ that takes value 0, and $\delta _{0}^{1}$ is 0 everywhere in ${\mathbb {Z}}^{2}$ except that it takes value 1 at $0\in {\mathbb {Z}}^{2}$. Given a finite subset $D\subseteq {\mathbb {Z}}^{2},$ denote by ${\ell }_{\mathrm{TS}}(D)$ the length of a shortest path in ${\mathbb {Z}}^{2}$ (with respect to the standard generating set $\left\{ e_{1},e_{2}\right\}$) that visits every point in *D*. One of the usual settings for the TSP also specifies the beginning and ending points of the path. Here we do not specify the beginning and ending points (in the situations we consider the diameter of *D* is much smaller than $\ell _{\mathrm{TS}}(D)$). Then the word length of a group element $g=(x,f)$ satisfies

$$\begin{aligned} {{\ell }_{\mathrm{TS}}}\left( \mathrm{supp}f\right) +|\mathrm{supp}f|\le l_{S}(g)\le {{\ell }_{\mathrm{TS}}}\left( \mathrm{supp}f\right) +|\mathrm{supp}f|+|x|+2\mathrm{Diam}(\mathrm{supp}f), \end{aligned}$$

where $\left| X\right|$ is the cardinality of a set *X*, and $\mathrm{supp}f=\{x\in {\mathbb {Z}}^{2}:f(x)\ne 0\}$.

Consider the standard switch-walk-switch (SWS) measure $\mu =\eta *\nu *\eta$ on ${\mathbb {Z}}^{2}\wr \left( {\mathbb {Z}}/2{\mathbb {Z}}\right)$, where $\eta$ is uniform on $\left\{ id_{G},\delta \right\}$ and $\nu$ is uniform on $\left\{ s_{1}^{\pm 1},s_{2}^{\pm 1}\right\}$. We refer to the random walk $\left( X_{n}\right) _{n=0}^{\infty }$ with step distribution $\mu$ as the standard SWS random walk on ${\mathbb {Z}}^{2}\wr \left( {\mathbb {Z}}/2{\mathbb {Z}}\right)$. Write $X_{n}=\left( {\bar{X}}_{n},\Phi _{n}\right)$. Denote by ${\mathcal {R}}_{n}$ the range of the projected random walk $\left( {\bar{X}}_{n}\right) _{n=0}^{\infty }$ on ${\mathbb {Z}}^{2}$ up to time *n*. It is easy to see that $l_{S}(X_{n})$ is equivalent to the size of the range ${\mathcal {R}}_{n}$, up to a multiplicative constant. Our goal is to determine the asymptotics of $l_{S}(X_{n})/(n/\log n)$ when $n\rightarrow \infty$. Given a parameter $p\in (0,1)$, the set obtained by keeping each point in ${\mathcal {R}}_{n}$ independently with probability *p* is called the diluted range with parameter *p*. Then the distribution of $\mathrm{supp}\Phi _{n}$ of the standard SWS random walk is the same as the diluted range ${\mathcal {R}}_{n}$ with parameter 1/2. Therefore we are led to consider the $\mathrm{TSP}$ of the diluted range.

Let $F_{n}$ be the length of a shortest path that visits the sites of a diluted square two-dimensional lattice of side length *n* with parameter *p*, then by subadditivity, ${\mathbb {E}}[F_{n}]/n^{2}$ converges to a constant $\alpha _{p}$ when $n\rightarrow \infty$, see Lemma 2.3. Indeed, the stronger statement that $F_{n}/n^{2}$ converges to $\alpha _{p}$ almost surely is true: this observation probably goes back to Beardwood et al. [4]. The TSP on diluted lattice was considered by Chakrabarti [14] and Dhar et al. [19] on the triangular lattice and the square lattice, who estimated the constant $\alpha _{p}$ in terms of the percolation parameter *p*.

We estimate the length of $\mathrm{TSP}$ of the diluted range, claiming that this length, normalized by the size of the range, converges almost surely to a positive constant, see Lemma 2.7. To show this claim we subdivide ${\mathbb {Z}}^{2}$ into boxes of certain side length *C* and inside each each box use the uncrossing Lemma 2.5. Part (i) of Lemma 2.5 is similar the circle freeway lemma used by Lalley in [33] for the TSP of random points on ${\mathbb {R}}^{2}$ with self-similar distribution; and in part (ii) we estimate the number of “ uncrossings”. To control the error of approximations, we rely on the Følner property of the range process $\left( {\mathcal {R}}_{n}\right) _{n=1}^{\infty }$. Given a finite $V\subset {\mathbb {Z}}^{2}$, denote by $\partial V$ the inner boundary of *V*, that is, the set of points in *V* which have at least one neighbor site outside *V*. We say that a sequence of finite sets $\left( V_{n}\right) _{n=1}^{\infty }$ forms a a Følner sequence if $\left| \partial V_{n}\right| /\left| V_{n}\right| \rightarrow 0$ as $n\rightarrow \infty$. Consider the inner boundary $\partial {\mathcal {R}}_{n}$ of the random walk range ${\mathcal {R}}_{n}$. In Okada [41] it is shown that there exists a constant $c\in \left[ \pi ^{2}/2,2\pi ^{2}\right]$ such that $\lim _{n\rightarrow \infty }\frac{{\mathbb {E}}\left[ \left| \partial {\mathcal {R}}_{n}\right| \right] }{n/\log ^{2}n}=c$. The property that almost surely $\left( {\mathcal {R}}_{n}\right) _{n=1}^{\infty }$ forms a Følner sequence is proved for a finite range symmetric random walk on ${\mathbb {Z}}^{2}$ in Deligiannidis and Kosloff [17]; and extended to symmetric random walks on ${\mathbb {Z}}^{2}$ with finite second moment in Deligiannidis et al. [16]. More precisely, by [16, Theorem 12], for a centered, non-degenerate random walk of finite second moment on ${\mathbb {Z}}^{2}$, there exists a constant $c\in (0,\infty )$ such that almost surely

$$\begin{aligned} \lim _{n\rightarrow \infty }\frac{\left| \partial {\mathcal {R}}_{n}\right| }{n/\log ^{2}n}=c. \end{aligned}$$

The proof for general random walks as in the statement of Theorem 1.1, not necessarily SWS random walks, follows a similar outline, and we explain below additional arguments we have to use in this general case. We need in particular to have an analog of a “ simple” lemma for values of configurations in the wreath product, rather than for diluted squares (see Lemma 3.1); and a more general form of “ the Uncrossing Lemma” in terms of wreath products (see Lemma 3.3). We also need to control the closeness of the values of the configuration to the i.i.d. on the range, see Lemma 4.2, in this step we use a result of Flatto [25]. The proof of the main result of that paper is written for simple random walk, but the proof works for centered random walks with $(2+\epsilon )$-moment, see [25, Remark after Theorem 3.1]. This is the only place we need the finite $(2+\epsilon )$-moment assumption rather than finite second moment. It is natural to ask whether the statement of [25] is true under finite second moment assumption, but to our knowledge it is not known. Finally, we mention one more extra ingredient what we need for the proof of Theorem 1.1 in the case when the measure is infinitely supported. We need to control large jumps between visited squares of subdivision, see the proof of upper bound for $\left| X_{n}\right|$ at the end of Sect. 4 (here the assumption of finite second moment is sufficient).

## Proof of theorem 1.1 in the case of standard SWS random walk and standard generating set

In this section we consider the case where *S* is the standard generating set of $G={\mathbb {Z}}^{2}\wr \left( {\mathbb {Z}}/2{\mathbb {Z}}\right)$, $S=\left\{ s_{1}=(e_{1},\mathbf{0}),s_{2}=(e_{2},\mathbf{0}),\gamma =(0,\delta _{0}^{1})\right\}$; as we have mentioned in the introduction, $e_{1},e_{2}$ are standard generators of ${\mathbb {Z}}^{2}$ and $\delta _{0}^{1}:{\mathbb {Z}}^{2}\rightarrow {\mathbb {Z}}/2{\mathbb {Z}}$ is the delta function at (0, 0), defined by $\delta _{0}^{1}(0,0)=1$ and $\delta _{0}^{1}(x)=1$ if $x\ne (0,0)$. We consider random walk step distribution $\mu$ is the switch-walk-switch measure $\eta *\nu *\eta$, where $\eta$ is uniform on $\left\{ id_{G},\delta \right\}$ and $\nu$ is uniform on $\left\{ (\pm e_{1},\mathbf{0}),(\pm e_{2},\mathbf{0})\right\}$. The projection of $\mu$ to ${\mathbb {Z}}^{2}$ is $\nu$, the standard simple random walk on ${\mathbb {Z}}^{2}$.

We recall the notation for the boundary of set *V*. Given a finite set $V\subset {\mathbb {Z}}^{2}$, denote by $\partial V$ the inner boundary of *V*, that is, the set of points in *V* which have at least one neighbor site outside *V*.

### First observations about range of simple random walks on ${\mathbb {Z}}^{2}$

Given a finite set $V\subseteq {\mathbb {Z}}^{2}$, recall that we denote by $l_{\mathrm{TS}}(V)$ the length of a shortest path in the Cayley graph of ${\mathbb {Z}}^{2}$ with respect to generators $\{e_{1}$, $e_{2}\}$ that visits all vertices in *V*. Note the following property of connected sets in ${\mathbb {Z}}^{2}$.

#### Lemma 2.1

Let *V* be a connected subset of ${\mathbb {Z}}^{2},$then

$$\begin{aligned} {\ell _{\mathrm{TS}}}(V)\le |V|\left( 1+8\left( \frac{|\partial V|}{|V|}\right) ^{1/3}\right) . \end{aligned}$$

#### Proof

For any positive integer *C*, subdivide ${\mathbb {Z}}^{2}$ into squares of size $C\times C$. Consider only those squares that intersect with *V* and denote by *T* the union of the perimeters of these squares. Denote by $V'$ the subset of *V* which consists of points whose squares are not completely contained in *V*. Note that since by assumption *V* is a connected subset of ${\mathbb {Z}}^{2}$, we have that *T* is connected as well. We have that

$$\begin{aligned} \left| T\right|&\le 4C\left( \frac{\left| V\right| }{C^{2}}+\left| \partial V\right| \right) ,\\ \left| V'\right|&\le C^{2}\left| \partial V\right| . \end{aligned}$$

Let $\gamma _{\mathrm{TS}}(T)$ be a shortest path that goes through all edges in *T*. One can then visit all points in *V* by going along this path through *T* in the following way. We follow the path $\gamma _{\mathrm{TS}}(T)$ that visits points of *T*. For each $C\times C$ square *S*, look at the first visit of its boundary by $\gamma _{\mathrm{TS}}(T)$. We stop at this point, insert a path that visits all points of $V\cap S$, return along *T* to the same point of the boundary of *S* where the path inside *S* is inserted, and then continue along the path $\gamma _{\mathrm{TS}}(T)$. Such path provides an upper bound for ${\ell _{\mathrm{TS}}}(V$

$$\begin{aligned} {\ell _{\mathrm{TS}}}(V)&\le \left| \gamma _{\mathrm{TS}}(T)\right| +\left( 4C+C^{2}\right) \frac{\left| V\right| }{C^{2}}+2|V'|\\&\le |V|+8\frac{\left| V\right| }{C}+\left( 4C+2C^{2}\right) |\partial V|. \end{aligned}$$

Choosing that $C>0$ such that $\left( C+1\right) ^{3}=4|V|/\left| \partial V\right|$, we obtain the statement of lemma. $\square$

For the $\nu$-random walk on ${\mathbb {Z}}^{2}$, by [17, Theorem 4.1], the sequence $\left( {\mathcal {R}}_{n}\right) _{n=1}^{\infty }$ is almost surely a Følner sequence. Thus we have the following:

#### Corollary 2.2

Consider a standard simple random walk on ${\mathbb {Z}}^{2}$. Let ${\mathcal {R}}_{n}$ be range of the random walk up to time *n*, then

$$\begin{aligned} \lim _{n\rightarrow \infty }\frac{\ell _{\mathrm{TS}}\left( {\mathcal {R}}_{n}\right) }{\left| {\mathcal {R}}_{n}\right| }=1 \text{ a.s. } \end{aligned}$$

#### Proof

By [17, Theorem 4.1], $\left| \partial R_{n}\right| /\left| R_{n}\right| \rightarrow 0$ a.s. when $n\rightarrow \infty$, thus for ${\mathbb {P}}$-a.e. $\omega$, there exists a number $N(\omega )=N_{\epsilon }(\omega )$ such that for any $n\ge N$, we have $\left| \partial R_{n}\right| /|R_{n}|<\epsilon$. It follows that from Lemma 2.1, for $n>N(\omega )$, $R_{n}^{\mathrm{TSP}}\le (1+8\epsilon ^{1/3})|R_{n}|$. In the lower bound direction, $R_{n}^{\mathrm{TSP}}\ge |R_{n}|$, the statement follows. $\square$

### An auxiliary fact about TSP on the diluted lattices

Consider a square of size $n\times n$ in ${\mathbb {Z}}^{2}$ and denote by $D_{n}$ the diluted lattice (site percolation) where each vertex of the lattice is present independently with probability *p*. By almost sub-additivity we have the following.

#### Lemma 2.3

There exists a constant $\alpha _{p}>0$ such that ${\mathbb {E}}\left[ \ell _{\mathrm{TS}}\left( D_{n}\right) \right] /n^{2}\rightarrow \alpha _{p}$ when $n\rightarrow \infty$.

#### Proof

We first show the convergence along the subsequence $\left( 2^{m}\right) _{m=1}^{\infty }$. Consider a square of size $2^{m+1}\times 2^{m+1}$ and subdivide it into 4 squares $C_1$, $C_2$, $C_3$, $C_4$.. In each square $C_{i}$, $1 \le i \le 4$, take a traveling salesman path $P_{i}$ visiting the points in the diluted lattice in $C_{i}$. We denote by $A_{i}$ and $B_{i}$ the starting and ending point of $P_{i}$. Then one can find a path visiting all points in the diluted lattice of the larger square by following the paths $P_{1},P_{2},P_{3}$ and $P_{4}$, adding in shortest paths connecting $B_{1}$ to $A_{2}$, $B_{2}$ to $A_{3}$ and $B_{3}$ to $A_{4}$. Therefore

$$\begin{aligned} {\mathbb {E}}\left[ \ell _{\mathrm{TS}}\left( D_{2^{m+1}}\right) \right] \le 4{\mathbb {E}}\left[ \ell _{\mathrm{TS}}\left( D_{2^{m}}\right) \right] +9\cdot 2^{m}. \end{aligned}$$

Write $b_{m}={\mathbb {E}}\left[ \ell _{\mathrm{TS}}\left( D_{2^{m}}\right) \right] /2^{2m}$, then we have that $b_{m+1}\le b_{m}+9\cdot 2^{-m-2}$. It follows that the sequence $b_{m}$ converges to a limit constant $\alpha _{p}$. Since ${\mathbb {E}}\left[ \left| D_{2^{m}}\right| \right] \ge p2^{2m}$, we have that $\alpha _{p}\ge p>0$.

For general *n*, take $2^{j}$ such that $2^{j}\ll n$. Consider the subsquare of side length $2^{j}\left\lfloor n/2^{j}\right\rfloor$ inside the square of side length *n* with the same lower left corner. Then we have

2.1 $$\begin{aligned} {\mathbb {E}}\left[ \ell _{\mathrm{TS}}\left( D_{n}\right) \right] \le {\mathbb {E}}\left[ \ell _{\mathrm{TS}}\left( D_{2^{j}\left\lfloor n/2^{j}\right\rfloor }\right) \right] +4\cdot 2^{j}n. \end{aligned}$$

To get a lower bound, for $2^{k}\gg n$, divide its sub-square of side length $n\left\lfloor 2^{k}/n\right\rfloor$ into squares of side length *n* and connecting the TSP inside each small square, we have

2.2 $$\begin{aligned} {\mathbb {E}}\left[ \ell _{\mathrm{TS}}\left( D_{2^{k}}\right) \right] \le \left( \frac{2^{k}}{n}\right) ^{2}{\mathbb {E}}\left[ \ell _{\mathrm{TS}}\left( D_{n}\right) \right] +C2^{2k}/n. \end{aligned}$$

It follows that ${\mathbb {E}}\left[ \ell _{\mathrm{TS}}\left( D_{n}\right) \right] /n^{2}$ converges to $\alpha _{p}$ as $n\rightarrow \infty$. $\square$

#### Remark 2.4

It is not hard to see that $p<\alpha _{p}<1$. Indeed, observe that a positive frequency of absent squares of size $C\times C$, for a large constant *C*, implies that $\alpha _{p}<1$. On the other hand, positive frequencies of hanging edges show that $\alpha _{p}>p$. The Bartholdi–Platzman bound from [3] (which is recalled in Lemma 4.3) implies that $\lim _{p\rightarrow 0+}\alpha _{p}=0$.

### An uncrossing lemma

In this subsection, different from the discrete setting of the previous subsection, we consider domains and paths in the plane. The Cayley graph of ${\mathbb {Z}}^2$ with respect to the standard generators $\{e_1,e_2\}$ is naturally embedded as a lattice in the plane. A key ingredient in our argument is the following uncrossing lemma for paths in the plane.

Let *D* be a bounded domain (a non-empty, connected and open set ) in the plane. Denote by $\partial D$ the complement of *D* in its closure ${\bar{D}}$. (Note that this notation is not the same as the definition of $\partial V$ for a subset *V* in a graph.) We assume that the boundary $\partial D$ is a simple, closed, rectifiable curve. Consider two paths $P_{1}$, $P_{2}$ where each $P_{i}$ starts and terminates at the boundary $\partial D$, write $A_{i}$ for the starting point of $P_{i}$ and $B_{i}$ for the end point of $P_{i}$. We say $P_{1}$ and $P_{2}$ have an *essential crossing* if $A_{2}$ and $B_{2}$ are contained in the two different open arcs of $\partial D$ between $A_{1}$ and $B_{1}.$

Given a finite collection ${\mathcal {T}}$ of paths starting and ending at the boundary $\partial D$, we perform the following procedure. If there are two paths $P_{1}$ and $P_{2}$ with a common starting/ending point such that $\left\{ A_{1},B_{1}\right\} \cap \left\{ A_{2}, B_{2}\right\} \ne \emptyset$, then reversing the direction of one of them if necessary, we replace them by the path which is the concatenation of $P_{1},P_{2}$. By performing this repeatedly, we can replace ${\mathcal {T}}$ by ${\mathcal {T}}'$ in such a way that the starting and ending points of the paths in the new collection ${\mathcal {T}}'$ are pairwise disjoint. In ${\mathcal {T}}'$ each starting or ending point appears exactly once, unless there are paths with the same starting/ending point (these are loops based at $\partial D$). We keep the loops as they are. Note that by definition, a loop does not have essential crossing with any other path.

**Fig. 2:**
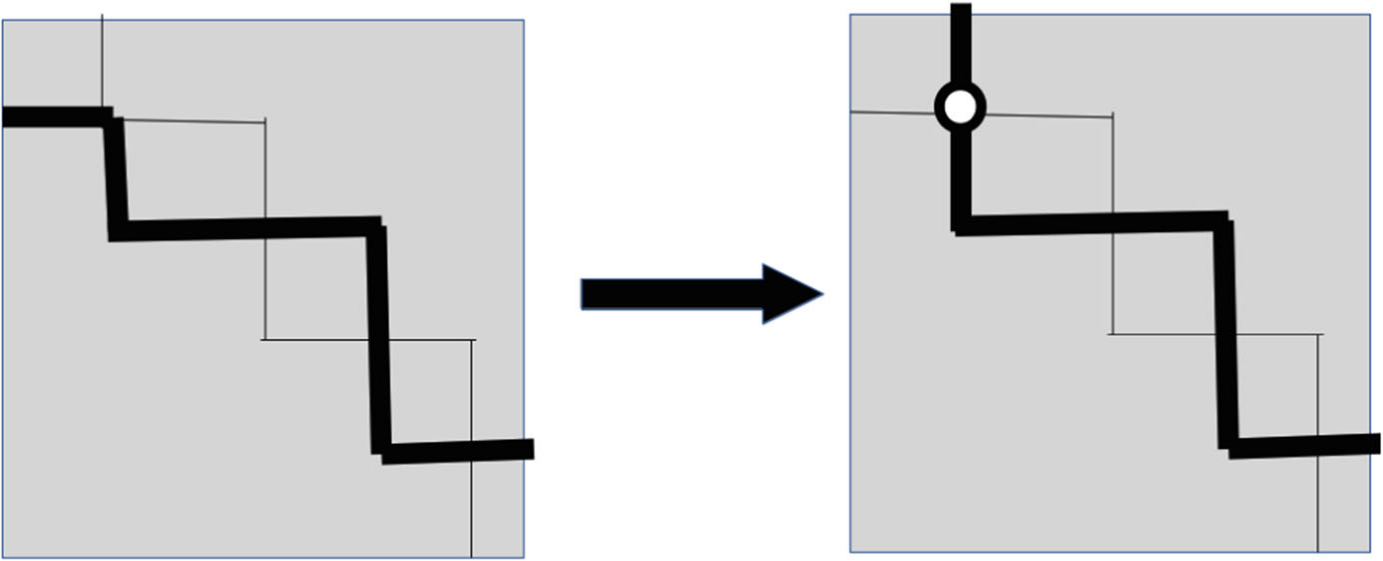
Uncrossing two paths

By continuity, if two paths $P_{1}$ and $P_{2}$ have essential crossings then they must intersect. Let *O* be an intersection point of $P_{1}$ and $P_{2}$. We call the following operation *uncrossing of* $P_{1}$ *and* $P_{2}$ *around* *O*: replace the path $P_{1}$ by $Q_{1}$, where $Q_{1}$ is the concatenation of the segment of $P_{1}$ from $A_{1}$ to *O* and the segment of $P_{2}$ from *O* to $B_{2}$; and similarly replace $P_{2}$ by $Q_{2}$ which concatenates the segment of $P_{2}$ from $A_{2}$ to *O* and the segment of $P_{1}$ from *O* to $B_{1}$. It is clear that the union of images of $Q_{1}$ and $Q_{2}$ is the same as the union of $P_{1}$ and $P_{2}$, and, moreover, $Q_{1}$ and $Q_{2}$ do not have an essential crossing. See Fig. 2. Note that $Q_{1}$ and $Q_{2}$ might have intersection points other than *O*.

Apply this uncrossing procedure repeatedly, we have the following lemma. The first part is essentially the same as *the circle freeway lemma* in Lalley [33].

#### Lemma 2.5

(Uncrossing lemma). Suppose *D* is a bounded domain in the plane whose boundary $\partial D$ is a simple, closed, rectifiable curve. Consider a collection ${\mathcal {T}}$ of paths in *D* where each path starts and ends on the boundary of *D*.

**(i)**: There is another collection ${\mathcal {S}}$ of paths, which has the same union of their images as the initial one, and no two paths in the new collection has an essential crossing.
**(ii)**: One can obtain such a collection ${\mathcal {S}}$ from the original collection ${\mathcal {T}}$ by first replacing ${\mathcal {T}}$ by ${\mathcal {T}}'$ such that the paths in ${\mathcal {T}}'$ have pairwise disjoint starting and ending points, and then performing at most *m* uncrossings, where *m* is the number of paths in ${\mathcal {T}}'$ which are not loops.

#### Proof

Given a finite collection of paths ${\mathcal {T}}$ in *D*, we first replace ${\mathcal {T}}$ by ${\mathcal {T}}'$ via joining paths (reversing direction of some of them if necessary) as explained before.

We prove the statement by induction on the number of paths of in the collection ${\mathcal {T}}'$. Given a path *P* with starting point *A* and ending point *B*, denote by $C_{1},C_{2}$ the two arcs on $\partial D$ between *A* and *B*. We say that a path *P* in the collection ${\mathcal {T}}'$ is good if there exists *i*, $i=1$ or $i=2$, such that all other paths in ${\mathcal {T}}'$ have starting and ending points in $C_{i}$. In particular it implies that *P* has no essential crossings with the other paths. If there is a good path *P* in ${\mathcal {T}}',$ then we can remove it and apply the induction hypothesis to the collection ${\mathcal {T}}'{\setminus }\{P\}$ consisting of the rest of paths. Note that performing uncrossing for the rest of the paths will not introduce any essential crossing with *P*. If there is no good path, pick any path *P* and on the arc $C_{1}$ take the point $A'$ which is the closest to *A* which is starting or ending point some other path *Q* in the collection. Reverse the direction of *Q* if necessary, we uncross *P* and *Q* such that one of the new path starts at *A* and end at $A'$. Now this new path is good, we remove it and apply the induction hypothesis. $\square$

After performing uncrossings to the original collection ${\mathcal {T}}$, we can join the paths in the new collection ${\mathcal {S}}$. We use the notation $\left| P\right|$ for the length of a curve *P*.

#### Lemma 2.6

Suppose ${\mathcal {S}}$ is a collection of paths in the domain *D* such that each path starts and ends on $\partial D$ and there is no essential crossings between paths of ${\mathcal {S}}$. Then there is a path of total length at most $1.5\left| \partial D\right| +\sum _{P\in {\mathcal {S}}}|P|$ whose image is obtained from the union of paths in ${\mathcal {S}}$ by adding connecting segments along the perimeter of *D*.

**Fig. 3:**
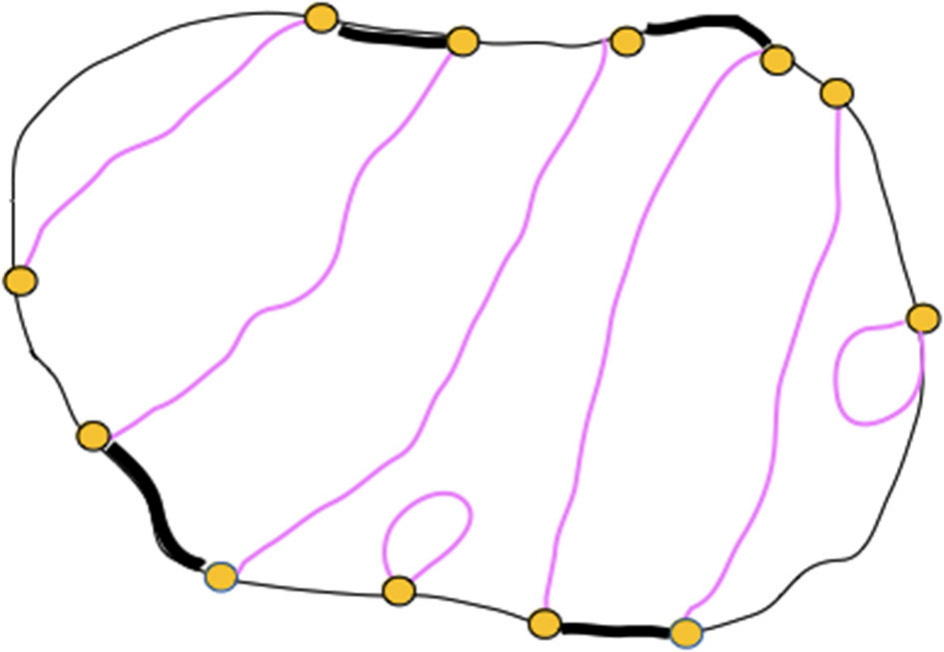
First we ignore possible loops, pass through all other paths in the collection, adding intervals on the perimeter of the domain (shown in black). The total sum of these black intervals can be chosen to be at most one half of the perimeter

#### Proof

First we pass through all paths that are not loops (see Fig. 3). The length of this path is at most the sum of the lengths of non-loop paths plus $|\partial D|/2$. Then we go through all the loops, passing through any point of the perimeter at most once. It is clear that the total sum of the segments of the perimeter we passed through is at most the length of the perimeter. Therefore the total length of our path is at most sum of the lengths of initial paths plus $1.5|\partial D|$. $\square$

The multiplicative constant 1.5 in front of $\left| \partial D\right|$ is not important for our applications, but it is optimal in the setting of Lemma 2.6.

We will apply the uncrossing lemma in the following context. We consider the standard generating set $\{e_{1},e_{2}\}$ of ${\mathbb {Z}}^{2}$, and the corresponding Cayley graph is naturally embedded in the plane. By a path in ${\mathbb {Z}}^{2}$ we mean a directed path in the standard Cayley graph of $\left( {\mathbb {Z}}^{2},(e_{1},e_{2})\right)$. Take a square *D* in the Cayley graph, then the vertex boundary of *D* is connected by a simple closed path in the Cayley graph of $\left( {\mathbb {Z}}^{2},(e_{1},e_{2})\right)$. With slight abuse of notation, we use the same notation $\partial D$ for this simple closed path.

### Standard random walk on ${\mathbb {Z}}^{2}\wr ({\mathbb {Z}}/2{\mathbb {Z}})$

**Fig. 4:**
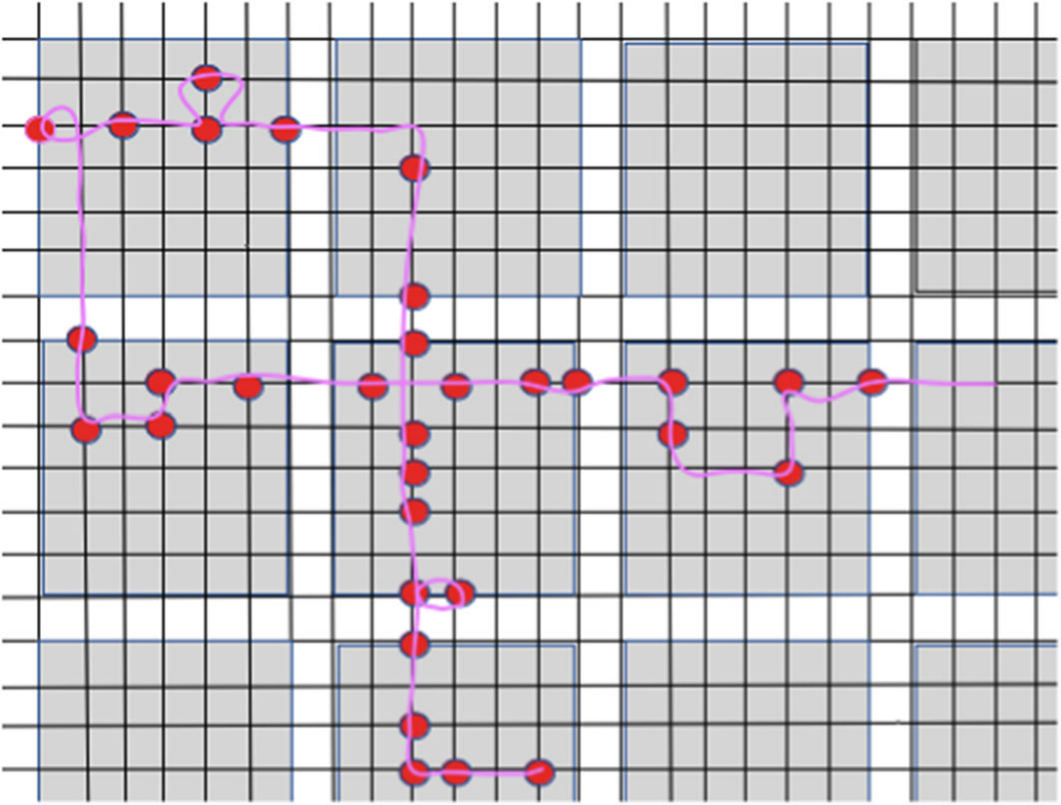
We subdive a path which visits the red nodes into intervals that stay inside each grey square of side length *C* (color figure online)

**Fig. 5:**
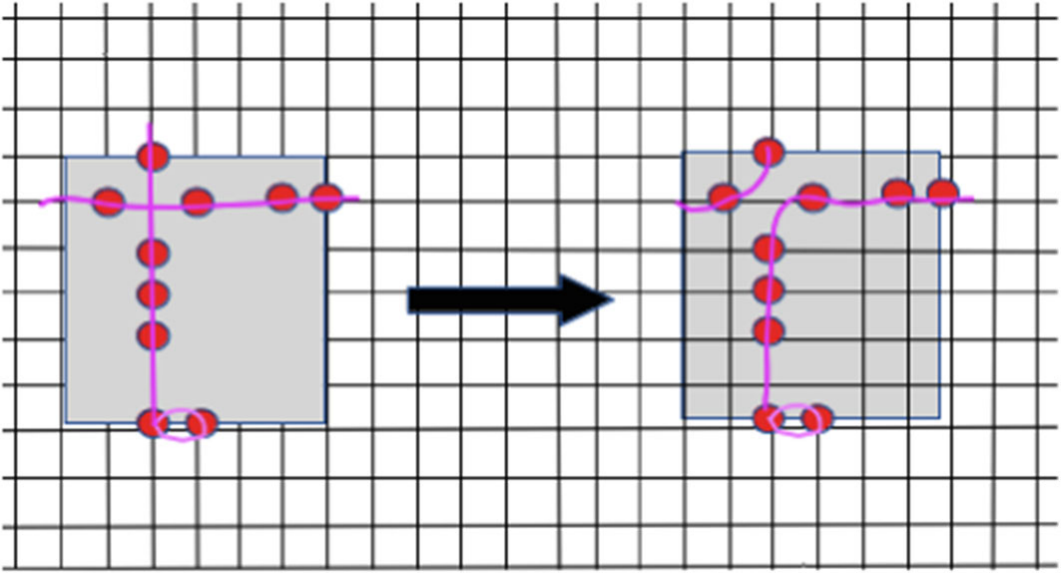
Given a collection of paths inside a grey square, we perform the uncrossing procedure

**Fig. 6:**
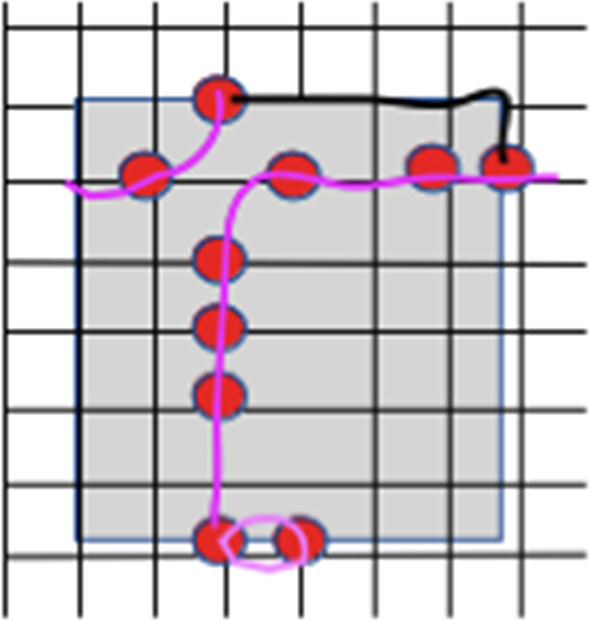
Given a collection of (non-oriented) paths (magenta coloured paths on the picture) without essential crossings, we join them together in one single path, adding new paths in the boundary of the square (on this picture there is only one additional path, shown in black). The absence of essential uncrossings guarantees that the total length of new paths is at most the perimeter of the square (that is, at most 4C) (color figure online)

Denote by $\mathcal {DR}_{n}$ the diluted range ${\mathcal {R}}_{n}$ with parameter 1/2. Recall that $\ell _{\mathrm{TS}}(\mathcal {DR}_{n})$ denotes the shortest length of a path visiting all points in the diluted range $\mathcal {DR}_{n}$. By using the Uncrossing Lemma 2.5, we show that $\ell _{\mathrm{TS}}(\mathcal {DR}_{n})/|{\mathcal {R}}_{n}|$ converges to a positive constant almost surely. The steps in the proof are illustrated in Figs. 4, 5 and 6.

#### Lemma 2.7

The TSP length of the diluted range satisfies

$$\begin{aligned} \frac{\ell _{\mathrm{TS}}(\mathcal {DR}_{n})}{|{\mathcal {R}}_{n}|}\rightarrow \alpha _{1/2} \text{ a.s. } \text{ when } n\rightarrow \infty , \end{aligned}$$

where the constant $\alpha _{1/2}$ is the same as in Lemma 2.3 with $p=1/2$.

#### Proof

Take a large integer *C* and divide ${\mathbb {Z}}^{2}$ into boxes of side length *C*. Consider those boxes with non-empty intersection with the range ${\mathcal {R}}_{n}$.

We first show an upper bound on $\ell _{\mathrm{TS}}(\mathcal {DR}_{n})$ by describing a path that visits all points in $\mathcal {DR}_{n}$. Take a path that consists of the union $T_{n}$ of the perimeters of the boxes with non-empty intersection with ${\mathcal {R}}_{n}$. We say a box $\alpha$ is fully visited if $\alpha \subseteq {\mathcal {R}}_{n}$. Suppose $\alpha$ is a box not full, we take a path that starts at a point of $\alpha \cap \partial {\mathcal {R}}_{n}$, visits all points of ${\mathcal {R}}_{n}\cap \alpha$ and returns to its staring point. The length of such path is at most $2|{\mathcal {R}}_{n}\cap \alpha |$. If a box $\alpha$ is full, then inside $\alpha$ we are in the situation of TSP of a diluted lattice. Therefore, to visit all points in ${\mathcal {D}}{\mathcal {R}}_{n}$, we can go along the perimeter $T_{n}$, and in each box $\alpha$ we take a shortest path visiting all points inside $\mathcal {DR}_{n}|_{\alpha }$ then return to $T_{n}$. The length of such a path is bounded by

2.3 $$\begin{aligned} \ell _{\mathrm{TS}}(\mathcal {DR}_{n})\le \sum _{\alpha \text{ full }}\ell _{\mathrm {TS}}\left( \mathcal {DR}_{n}|_{\alpha }\right) +\sum _{\alpha \ \text{ not } \text{ full }}2|{\mathcal {R}}_{n}\cap \alpha |+2|T_{n}|. \end{aligned}$$

If a box is not full, then it must contain some point in $\partial {\mathcal {R}}_{n}$. Therefore the number of boxes which are not full is bounded by $\left| \partial {\mathcal {R}}_{n}\right|$ and

$$\begin{aligned} \sum _{\alpha \ \text{ not } \text{ full }}|{\mathcal {R}}_{n}\cap \alpha |\le C^{2}|\partial {\mathcal {R}}_{n}|. \end{aligned}$$

Similarly the length of the perimeter union $T_{n}$ is bounded by

$$\begin{aligned} |T_{n}|\le 4|\partial {\mathcal {R}}_{n}|+4\left| {\mathcal {R}}_{n}\right| /C. \end{aligned}$$

Plugging these bounds into (2.3), we obtain

2.4 $$\begin{aligned} {\ell _{\mathrm{TS}}}\left( \mathcal {DR}_{n}\right) \le \sum _{\alpha \text{ full }}\mathrm{\ell _{\mathrm{TS}}}\left( \mathcal {DR}_{n}|_{\alpha }\right) +(2C^{2}+4)|\partial {\mathcal {R}}_{n}|+4|{\mathcal {R}}_{n}|/C. \end{aligned}$$

The main contribution to the length of the path comes from the TSP paths in full boxes. Denote by $D_{C}$ the diluted lattice obtained from a $C\times C$ square where each vertex of the square is present independently with probability 1/2. By the law of large numbers,

2.5 $$\begin{aligned} \frac{\sum _{\alpha \text{ full }}{\ell _{\mathrm{TS}}}\left( \mathcal {DR}_{n}|_{\alpha }\right) }{C^{2}\cdot \# \text{ of } \text{ full } \text{ boxes } \text{ in } {\mathcal {R}}_{n}}\rightarrow \frac{{\mathbb {E}}\left[ \ell _{\mathrm{TS}}\left( D_{C}\right) \right] }{C^{2}} \text{ a.s. } \text{ when } n\rightarrow \infty . \end{aligned}$$

Now we turn to the lower bound. Take a global TSP path *P* in the standard generating set $\left\{ \pm e_{1},\pm e_{2}\right\}$ of ${\mathbb {Z}}^2$ which visits all the points in the diluted range ${\mathcal {D}}{\mathcal {R}}_{n}$. Consider a box $\alpha$ that is fully visited and restrict the path *P* to $\alpha$. We obtain a collection of connected paths where each path starts and ends on the boundary of $\alpha$. We now apply the Uncrossing Lemma 2.5 to this collection of paths and connect the uncrossed paths along the perimeter of $\alpha$, using Lemma 2.6. It follows that there is a connected path of length at most $\left| P|_{\alpha }\right| +6C$ which visits all points in $\mathcal {DR}_{n}|_{\alpha }$. Therefore, we have that for each fully visited box $\alpha$,

$$\begin{aligned} \left| P|_{\alpha }\right| \ge {\ell _{\mathrm{TS}}}\left( \left| P|_{\alpha }\right| \right) -6C. \end{aligned}$$

Summing up over all fully visited box, we have

2.6 $$\begin{aligned} {\ell _{\mathrm{TS}}}\left( \mathcal {DR}_{n}\right) \ge \sum _{\alpha \text{ full }}\left( {\ell _{\mathrm{TS}}}\left( \mathcal {DR}_{n}|_{\alpha }\right) -6C\right) . \end{aligned}$$

Finally we combine the upper and lower bounds. Given any $\epsilon >0$, by Lemma 2.3, we can take a constant $C>100/\epsilon$ sufficiently large such that

$$\begin{aligned} \left| \frac{{\mathbb {E}}\left[ \ell _{\mathrm {TS}}\left( D_{C}\right) \right] }{C^{2}}-\alpha _{1/2}\right| <\epsilon /100. \end{aligned}$$

Then by combining (2.4), (2.5) and (2.6) and applying the result that ${\mathcal {R}}_{n}$ forms a Følner sequence almost surely, we conclude that almost surely,

$$\begin{aligned} \limsup _{n\rightarrow \infty }\left| \frac{\mathrm{\ell _{TS}}(\mathcal {DR}_{n})}{|{\mathcal {R}}_{n}|}-\alpha _{1/2}\right| <\epsilon . \end{aligned}$$

Since $\epsilon >0$ is arbitrary, we have proved the statement. $\square$

#### Proof of Theorem 1.1 for standard SWS measure and standard generating set

Recall that $(X_{n})_{n=0}^{\infty }$ denotes a $\mu$-random walk on *G* and we write $X_{n}=\left( {\bar{X}}_{n},\Phi _{n}\right)$. The distribution of support of $\Phi _{n}$ is the same as the diluted range $\mathcal {DR}_{n}$ with parameter $p=1/2$. For the group element $X_{n}$, a path in $S\cup S^{-1}$ that connects $id_{G}$ to $X_{n}$ satisfies that its projection to the base ${\mathbb {Z}}^{2}$ visits all the points in $\mathrm{supp}\Phi _{n}$ and in the end arrives at ${\bar{X}}_{n}$; and along the way switch on the lamps in $\mathrm{supp}\Phi _{n}$. Therefore, we have that

$$\begin{aligned} {\ell _{\mathrm{TS}}}(\mathrm{supp}\Phi _{n})+|\mathrm{supp}\Phi _{n}|\le \ell _{S}(X_{n})\le {\ell _{\mathrm{TS}}}(\mathrm{supp}\Phi _{n})+|\mathrm{supp}\Phi _{n}|+3\max _{1\le t\le n}|{\bar{X}}_{t}|_{{\bar{S}}}. \end{aligned}$$

Recall that $\mathrm{supp}\Phi _{n}$ can be identified with $\mathcal {DR}_{n}$. Since $\max _{1\le t\le n}|{\bar{X}}_{t}|_{{\bar{S}}}/(n/\log n)\rightarrow 0$ almost surely when $n\rightarrow \infty$, by Lemma 2.7 and the Dvoretzky–Erdös law of large numbers for the range (1.1), we have

$$\begin{aligned} \lim _{n\rightarrow \infty }\frac{1}{n/\log n}\ell _{S}(X_{n})&=\lim _{n\rightarrow \infty }\frac{1}{n/\log n}{\ell _{\mathrm{TS}}}(\mathrm{supp}\Phi _{n})+\lim _{n\rightarrow \infty }\frac{1}{n/\log n}|\mathrm{supp}\Phi _{n}|.\\&=\left( \alpha _{1/2}+\frac{1}{2}\right) \pi . \end{aligned}$$

$$\square $$

## Uncrossing lemma for wreath product ${\mathbb {Z}}^{2}\wr ({\mathbb {Z}}/2{\mathbb {Z}})$ and general generating set

In this section we consider an arbitrary finite generating set *S* of $G={\mathbb {Z}}^{2}\wr ({\mathbb {Z}}/2{\mathbb {Z}})$. By an *S*-path we mean a path in the right Cayley graph of $\left( G,S\right)$, denote by $(x_{0},x_{1},\ldots ,x_{k}),$where each $x_{i}\in G$ and $x_{i-1}^{-1}x_{i}\in S$. Recall that an element in the wreath product is denoted by (*x*, *f*), where $x\in {\mathbb {Z}}^{2}$ and $f:{\mathbb {Z}}^{2}\rightarrow {\mathbb {Z}}/2{\mathbb {Z}}$ is a function of finite support. Since the lamp group is ${\mathbb {Z}}/2{\mathbb {Z}}$, the configuration *f* can be identified with the support of *f*, that is, the finite set where the value of *f* is 1. Given a finite set *U* in ${\mathbb {Z}}^{2}$, consider an *S*-path $(x_{0},x_{1},\ldots ,x_{k})$ in ${\mathbb {Z}}^{2}\wr ({\mathbb {Z}}/2{\mathbb {Z}})$ such that $x_{0}$ is of the form $\left( z,\mathbf{0}\right)$ and the support of the lamp configuration of $x_{k}$ is exactly *U*. Denote by ${\ell }_{\mathrm{TS}}^{(G,S)}(U)$ the length of the shortest *S*-path with this property.

Analogous to Lemma 2.3, we have the following convergence of expectation of length of traveling salesman *S*-paths after scaling for the diluted lattice in a square of side length *n*. Consider a square of size $n\times n$ in ${\mathbb {Z}}^{2}$ with the lower left corner at (0, 0). Take the diluted lattice $D_{n}$ with parameter *p* inside the square where each vertex of the lattice is present independently with probability *p*.

### Lemma 3.1

There exists a constant $\alpha _{p,S}>0$ such that ${\mathbb {E}}\left[ {\ell }_{\mathrm{TS}}^{(G,S)}(D_{n})\right] /n^{2}\rightarrow \alpha _{p,S}$ when $n\rightarrow \infty$.

### Proof

Write $F_{n}={\mathbb {E}}\left[ {\ell }_{\mathrm{TS}}^{(G,S)}(D_{n})\right]$. For a square of size $2^{n+1}\times 2^{n+1}$, subdivide it into 4 squares. In each sub-square $C_{i}$, take a shortest *S*-path $P_{i}$ where the end point $B_{i}$ of $P_{i}$ is such that $B_{i}=(b_{i},f_{i}),$ $\mathrm{supp}f_{i}$ is exactly the diluted lattice in $C_{i}$. Write $\left( a_{i},\mathbf{0}\right)$ for the starting point of $P_{i}$. Then one can find an *S*-path visiting all points in the diluted lattice of the original square by following the paths $P_{1},P_{2},P_{3}$ and $P_{4}$, joining their starting/ending points by the shortest *S*-paths connecting $(b_{1},\mathbf{0})$ to $(a_{2},\mathbf{0})$, $(b_{2},\mathbf{0})$ to $\left( a_{3},\mathbf{0}\right)$ and $(b_{3},\mathbf{0})$ to $(a_{4},\mathbf{0})$. Therefore

$$\begin{aligned} F_{2^{n+1}}\le 4F_{2^{n}}+C2^{n}, \end{aligned}$$

where the constant *C* only depends on *S*. Write $b_{n}=F_{2^{n+1}}/2^{2n}$, then $b_{n+1}\le b_{n}+C2^{-n-2}$. In particular, the sequence $b_{n}$ is almost monotone and therefore converges to a limit $\alpha _{p,{\bar{S}}}$. Since $F_{2^{n}}\ge {\mathbb {E}}\left[ \left| D_{n}\right| \right] =p2^{2n}$, we have that $\alpha _{p}\ge p>0$. To extend the convergence to general *n*, we argue in the same way to establish bounds of the form (2.1) and (2.2) as in Lemma 2.3. $\square$

Given an *S*-path $P=(x_{0},x_{1},\ldots ,x_{\ell })$, denote by $\tau _{P}$ the difference between lamp configurations of $x_{\ell }$ and $x_{0}$, that is, $\tau _{P}:=f_{x_{\ell }}-f_{x_{0}}$. Write $|P|=\ell$ for the length of the path *P*. Given a collection ${\mathcal {A}}$ of *S*-paths, we refer to the sum of configurations $\sum _{P\in {\mathcal {A}}}\tau _{p}$ as the lamp configuration produced by ${\mathcal {A}}$.

### Remark 3.2

The fact that the lamp group is ${\mathbb {Z}}/2{\mathbb {Z}}$ allows us to reverse the direction of paths. If we reverse the direction of *P* to the path $\overleftarrow{P}=(x_{\ell },x_{\ell -1},\ldots ,x_{1},x_{0})$, then the path $\overleftarrow{P}$ makes the same changes in the lamp configuration as *P*, while in the projection to ${\mathbb {Z}}^{2}$ the starting and ending points are swapped. This is only valid when the lamp group is ${\mathbb {Z}}/2{\mathbb {Z}}$.

Next we explain how to perform uncrossings to *S*-paths. Recall that in Sect. 2 we have defined essential crossings for standard paths in ${\mathbb {Z}}^{2}$. The notion can be extended to *S*-paths. Two *S*-paths are said to have *essential crossings* if their projections to ${\mathbb {Z}}^{2}$ have essential crossings. We say two ${\bar{S}}$-paths $P_{1}$ and $P_{2}$ have an essential crossing in *D*, where $P_{i}$ starts at $A_{i}$ and ends at $B_{i}$, if $A_{1},A_{2},B_{1},B_{2}\in \partial D$, and $A_{2}$ and $B_{2}$ are contained in the two different open arcs of $\partial D$ between $A_{1}$ and $B_{1}.$ Here $\partial D$ denotes the boundary of *D* with respect to the standard generating set $\{e_{1},e_{2}\}$.

The following lemma is a generalization of the uncrossing Lemma 2.5 to *S*-paths. Consider a configuration *f* with $\mathrm{supp}f=U$. Recall that for the standard generating set considered in Sect. 2, the length of the shortest path that turns on the prescribed configuration *f*, whose starting and ending point in ${\mathbb {Z}}^{2}$ are $z_{1}$ and $z_{2}$ respectively, is exactly the length of a traveling salesman path which starts at $z_{1}$, visits every point in $\mathrm{supp}f$, and ends at $z_{2}$, plus $\left| \mathrm{supp}f\right|$. And for the standard SWS random walk considered in Sect. 2, the configuration at time *n* is the diluted range $\mathcal {DR}_{n}$ with parameter 1/2. Now in the general case (as in the setting of Lemma 3.3 below) we need to control the shortest path to produce the configuration, where possible moves are associated to the generating set *S* (that is, using *S*-paths). For example, these moves can be of the following form: first jump on ${\mathbb {Z}}^{2}$ at distance 2 on the right, turn on the lamp at distance 5 above the marker on ${\mathbb {Z}}^{2},$ and then jump at distance 3 down. Any finite generating set *S* can be interpreted as such rules, with finitely many jumps and finitely many turnings on the lamps. Therefore the following lemma is formulated in terms of wreath products (since it uses *S*-paths) and gives the statement similar to Lemma 2.5 for an arbitrary generating set.

### Lemma 3.3

(Uncrossing lemma in terms of wreath product generators). Let *D* be an $C\times C$ square in ${\mathbb {Z}}^{2}$. Consider a collection ${\mathcal {A}}$ of *S*-paths where the projection of each path to ${\mathbb {Z}}^{2}$ starts and terminates on the boundary of *D*. Then one can find another collection ${\mathcal {B}}$ of *S*-paths such that there are no essential crossings between paths in ${\mathcal {B}}$, each path in ${\mathcal {B}}$ starts and terminates at $\partial D$, and ${\mathcal {B}}$ produces the same lamp configuration as ${\mathcal {A}}$. Moreover the total length of *S*-paths in ${\mathcal {B}}$ is bounded by

$$\begin{aligned} \sum _{Q\in {\mathcal {B}}}|Q|\le \sum _{P\in {\mathcal {A}}}|P|+c|\partial D|, \end{aligned}$$

where *c* is a constant only depending on *S*.

### Proof

Given an *S*-path $P=\left( x_{0},x_{1},\ldots ,x_{k}\right)$, where each increment $x_{i-1}^{-1}x_{i-1}\in S$, connect each pair $(x_{i-1},x_{i})$ by a shortest path in the standard generating set $\left\{ \delta ,\left( e_{1}^{\pm 1},\mathbf{0}\right) ,\left( e_{2}^{\pm 1},\mathbf{0}\right) \right\}$. Denote by ${\hat{P}}$ the new path in the standard generating set. Given two *S*-paths $P_{1}$ and $P_{2}$ in a domain *D*, where $P_{i}$ starts at $A_{i}$ and ends at $B_{i}$, we perform un uncrossing as follows. First take the paths ${\hat{P}}_{1}$ and ${\hat{P}}_{2}$ in the standard generating set described above associated with $P_{1}$ and $P_{2}$. If ${\hat{P}}_{1}$ and ${\hat{P}}_{2}$ have essential crossings, then we take an intersection point *O* and perform the uncrossing around *O* as described in Sect. 2.3. This way we obtain two paths without essential crossing: ${\hat{Q}}_{1}$ which is the concatenation of the sub-path of ${\hat{P}}_{1}$ from $A_{1}$ to *O* and the sub-path of ${\hat{P}}_{2}$ from *O* to $B_{2}$; and ${\hat{Q}}_{2}$ which is the concatenation of the sub-path of ${\hat{P}}_{2}$ from $A_{2}$ to *O* and the sub-path of ${\hat{P}}_{1}$ from *O* to $B_{1}$. Now we convert the paths ${\hat{Q}}_{1},\hat{Q_{2}}$ in the standard generating set back to ${\bar{S}}$-paths. If *O* lies on both $P_{1}$ and $P_{2}$, then let $Q_{1}$ be the concatenation of the sub-path of $P_{1}$ from $A_{1}$ to *O* and the sub-path of $P_{2}$ from *O* to $B_{2}$; and similarly for $Q_{2}.$ If *O* is not on $P_{1},$ then let $y_{1}$ be the closest point to *O* which belongs to the intersection of $P_{1}$ and the sub-path of ${\hat{P}}_{1}$ from $A_{1}$ to *O*. Similarly, let $z_{1}$ the closest point to *O* which belongs to the intersection of $P_{2}$ and the sub-path of ${\hat{P}}_{2}$ from *O* to $B_{2}$. Connect $y_{1}$ to $z_{1}$ by an *S*-path of shortest length. Then we define $Q_{1}$ to be the concatenation of the *S*-path from $A_{1}$ to $y_{1}$ along $P_{1}$, the *S*-path connecting $y_{1}$ to $z_{1}$ and the *S*-path from $z_{1}$ to $B_{2}$ along $P_{2}$. In the same manner we obtain an *S*-path $Q_{2}$ from $A_{2}$ to $B_{1}$.

By the description above, it is clear that $\tau _{Q_{1}}+\tau _{Q_{2}}=\tau _{P_{1}}+\tau _{P_{2}}$ and moreover

$$\begin{aligned} |Q_{1}|+|Q_{2}|\le |P_{1}|+|P_{2}|+c, \end{aligned}$$

where the additive constant *c* only depends on the generating set *S*.

Consider a finite collection ${\mathcal {A}}$ of *S*-paths whose projection to ${\mathbb {Z}}^{2}$ starts and ends at $\partial D$. If there are two paths $P_{1}$ and $P_{2}$ such that $\left\{ A_{1},B_{1}\right\} \cap \left\{ A_{2}.B_{2}\right\} \ne \emptyset$, then reversing the direction of one of them if necessary (this is allowed because the lamp group is ${\mathbb {Z}}/2{\mathbb {Z}},$ see Remark 3.2), we replace them by the path which is the concatenation of $P_{1},P_{2}$. By performing this repeatedly, we can replace ${\mathcal {A}}$ by ${\mathcal {A}}'$ such that the starting/ending points of the paths are pairwise disjoint. By the inductive argument in the proof of Lemma 2.5 (ii), the number of uncrossings we need to perform is bounded by $\left| \partial D\right|$. Since each uncrossing costs at most an additive constant *c*, this implies the statement of the lemma. $\square$

## The general case and proof of Theorem 1.1

Throughout this section, let $\mu$ be a symmetric non-degenerate probability measure on $G={\mathbb {Z}}^{2}\wr ({\mathbb {Z}}/2{\mathbb {Z}})$ with finite second moment; and *S* be a finite generating set of *G*. By raising $\mu$ to a convolution power if necessary, we may assume that $\mu (id)>0$ and $\mu (\delta )>0$, where $\delta =\left( \delta _{0}^{1},\mathbf{0}\right)$. Recall that $l_{S}$ denotes the word length with respect to the generating set *S*. Recall that one of the assumptions of Theorem 1.1 is that $\mu$ has $(2+\epsilon )$-moment for some $\epsilon >0$

Let $\pi$ be the projection $G\rightarrow {\mathbb {Z}}^{2}$. Denote by ${\bar{\mu }}$ the projection of $\mu$ to ${\mathbb {Z}}^{2}$. Recall that $\left( X_{n}\right) _{n=0}^{\infty }$ is a random walk with step distribution $\mu$ on *G* and we write $X_{n}=\left( {\bar{X}}_{n},\Phi _{n}\right)$, where ${\bar{X}}_{n}=\pi \left( X_{n}\right)$ is the projection to ${\mathbb {Z}}^{2}$ and $\Phi _{n}$ is the lamp configuration of $X_{n}.$

### Remark 4.1

A well-known argument of Kaimanovich and Vershik [31] implies that if the support of $\mu$ on $H \wr B$ belongs to $H \cup B$, and the projected random walk on *H* is transient, then the random walk has non-trivial Poisson boundary. For the wreath product $H\wr {\mathbb {Z}}/2{\mathbb {Z}}$, where the group *H* has at least cubic volume growth, then the Poisson boundary of simple random walk (and more generally any finite first moment random walk) has positive drift $l>0$. This follows from an argument of Kaimanovich and Vershik combined with characterization of Varopoulos [52] of recurrent groups. For exposition see e.g. [53], to our knowledge all known proofs of this result rely on polynomial growth theorem [26]. The second moment condition in the statement of Theorem 1.1 cannot be relaxed. Here we do not need to use the notion of the Poisson boundary, but as we have mentioned already, transience of the projected random walk on *H* implies linear range on *H* and hence linear lower bound for the drift function of $H\wr B$. For example, in ${\mathbb {Z}}^2\wr {\mathbb {Z}}/2{\mathbb {Z}}$, if we take $\mu$ to be of finite first moment such that the projection to ${\mathbb {Z}}^2$ is a transient random walk, then $L_\mu (n)$ grows linearly.

Under our assumptions, by [16, Theorem 12], the range process $\left( R_{n}\right) _{n=0}^{\infty }$ of the ${\bar{\mu }}$-random walk on ${\mathbb {Z}}^{2}$ forms a sequence of Følner sets almost surely: more precisely, there exists a constant $c>0$ such that

$$\begin{aligned} \lim _{n\rightarrow \infty }\frac{\left| \partial R_{n}\right| }{n/\log ^{2}n}=c \text{ a.s. } \end{aligned}$$

We proceed by subdividing the ${\mathbb {Z}}^{2}$-lattice into boxes of side length $c_{n}$, where $c_{n}$ is an integer depending on *n* (to be specified later). We say a $(c_{n}\times c_{n})$-box $\alpha$ is visited up to time instant *n* if is $\alpha \cap R_{n}\ne \emptyset$. Note that in a box $\alpha$ where every point has been visited by the ${\bar{\mu }}$-random walk up to time *n*, the distribution of the lamp configuration in $\alpha$ is in general not uniform on $\{0,1\}^{\alpha }$. Instead we use that if the box is visited enough times, then the distribution of the lamp configuration is close to uniform and the TSP of the configuration can be controlled. For this purpose we need the following two lemmas.

Write $a=\min \left\{ \mu (id),\mu (\delta )\right\}$ and $\mu =au+(1-a)\mu '$, where *u* is the uniform measure on $\{id,\delta \}$. Let $(Y_{n})_{n=1}^{\infty }$ be a sequence of i.i.d. Bernoulli random variables, with ${\mathbb {P}}(Y_{i}=1)=a$ and ${\mathbb {P}}\left( Y_{i}=0\right) =1-a$. Let $(Z_{n})_{n=1}^{\infty }$ be a sequence of a sequence of i.i.d. random variables with distribution $\mu '$ and $\left( U_{n}\right) _{n=1}^{\infty }$ be a sequence of i.i.d. random variables with distribution *u*. Then

$$\begin{aligned} {\tilde{Z}}_{n}=Z_{n}{} \mathbf{1}_{\{Y_{n}=0\}}+U_{n}\mathbf{1}_{\{Y_{n}=1\}} \end{aligned}$$

has distribution $\mu$. We think of the $\mu$-random walk increments sampled in this way. Write ${\tilde{W}}_{n}={\tilde{Z}}_{1}\ldots {\tilde{Z}}_{n}$. We say that a location $x\in {\mathbb {Z}}^{2}$ receives a good multiplication up to time instant *n* if there is a time $t\le n$ such that $\pi \left( {\tilde{W}}_{t-1}\right) =x$ and $Y_{t}=1$.

By deleting the steps with $Y_{n}=1$ in the sequence $\left( {\tilde{Z}}_{n}\right)$ we obtain a sequence of i.i.d. random variables with distribution $\mu '$. Denote by $\left( W'_{t}\right) _{t=0}^{\infty }$ the random walk on *G* associated with this sequence of increments. We say a box $\alpha$ is *q*-fully visited by the $\mu '$-walk by the time *n* if for every $x\in \alpha$,

$$\begin{aligned} \left| \left\{ t\le n:\pi \left( W_{t}'\right) =x\right\} \right| \ge q. \end{aligned}$$

Denote by $F_{n,q}^{\alpha }$ the event that the box $\alpha$ is *q*-fully visited by the $\mu '$-walk up to time instant *n*.

### Lemma 4.2

Given a $c_{n}\times c_{n}$ box $\alpha$, denote by $B_{n}^{\alpha }$ the set of vertices in $\alpha$ which did not have any good multiplication up to time *n*. For any $\epsilon >0$, there exists constants $\lambda _{1},\lambda _{2}>0$ depending only on $a=\min \{\mu (id),\mu (\delta )\}$ and $\epsilon$, such that

$$\begin{aligned} {\mathbb {P}}\left( \left\{ |B_{n}^{\alpha }|\ge (1+\epsilon )(1-a)^{q}|\alpha |\right\} \cap F_{(1-a-\epsilon )n,q}^{\alpha }\right) \le e^{-\lambda _{1}n}+e^{-\lambda _{2}|\alpha |}. \end{aligned}$$

### Proof

Let $(N_{i})_{i=1}^{\infty }$ be an i.i.d. sequence of random variables with distribution ${\mathbb {P}}(N_{i}=0)=1-a$, ${\mathbb {P}}(N_{i}=k)=a^{k}(1-a)$ for $k\in {\mathbb {N}}$, independent of the $\mu '$-random walk $\left( W_{t}'\right)$. Write $S_{n,\epsilon }=N_{1}+\ldots +N_{(1-a-\epsilon )n}+(1-\alpha -\epsilon )n$. Denote by $E_{n}$ the event that $S_{n,\epsilon }>n$. By the Chernoff bound we have that ${\mathbb {P}}(E_{n})\le e^{-\lambda _{1}n}$ for some constant $\lambda _1$ that only depends on *a*.

If the box $\alpha$ is *q*-fully visited by the $\mu '$-walk by the time $(1-\alpha -\epsilon )$, then at time $S_{n,\epsilon }$ for the walk $\left( {\tilde{W}}_{t}\right)$, the probability that a vertex *x* has not received a good multiplication is bounded from above by

$$\begin{aligned} {\mathbb {P}}\left( N_{1}+\ldots +N_{q}=0\right) =(1-a)^{q}. \end{aligned}$$

Therefore by the Chernoff bound, we have

$$\begin{aligned} {\mathbb {P}}\left( \left\{ B_{S_{n,\epsilon }}^{\alpha }\ge (1+\epsilon )(1-a)^{q}|\alpha |\right\} \cap F_{(1-a-\epsilon )n,q}^{\alpha }\right) \le e^{-\lambda _{2}|\alpha |}. \end{aligned}$$

$$\square $$

Given two configurations $\phi _{1},\phi _{2}\in \{0,1\}^{\alpha }$ in the box $\alpha$, denote by $d_{H}(\phi _{1},\phi _{2})$ the Hamming distance between them, that is, $d_{H}\left( \phi _{1},\phi _{2}\right) =\left| \left\{ x\in \alpha :\ \phi _{1}(x)\ne \phi _{2}(x)\right\} \right|$.

Note that if a site $x\in \alpha$ has received a good update before time *n*, then the lamp configuration at *x* is uniform on $\{0,1\}$ at time *n*; moreover the configurations at such sites are independent. The coupling is defined as: $\Psi _{n}$ takes the same value as $\Phi _n$ at a point in $\alpha$ which receives a good update before *n*; and at a point that does not receive a good update before time *n*, the value of $\Psi _{n}$ is independent uniform on $\{0,1\}$. Then by definitions we have $d_{H}\left( \Psi _{n},\Phi _{n}|_{\alpha }\right) \le |B_{n}^{\alpha }|$, where $B_n^{\alpha }$ is the set of vertices in $\alpha$ which has not received a good update up to time *n* as in Lemma 4.2.

To control TSP of the configuration, we need the following classical bound due to Bartholdi and Platzman [3] (which they prove by space-filling curves heuristics; similar multiscale bound for TSP works for general graphs other than ${\mathbb {Z}}^{2}$, see e.g., the inequality (17) in [40]).

### Lemma 4.3

(Bartholdi–Platzman [3]). Let *A* be a set of *N* points in a square of side length *M* in ${\mathbb {Z}}^{2}$. Then $\ell _\mathrm{{TS}} (A) \le 2\sqrt{NM^{2}}$.

The rest of this section is devoted to the proof of Theorem 1.1.

### Proof of Theorem 1.1

Recall that we write $a=\min \left\{ \mu (id),\mu (\delta )\right\}$ and $\mu =au+(1-a)\mu '$, where *u* is the uniform measure on $\{id,\delta \}$. For any $\epsilon >0$, take *q* sufficiently large such that $10(1-a)^{q}<\epsilon$.

Divide ${\mathbb {Z}}^{2}$ by boxes of side length $c_{n}$. We first apply the result from [16] that almost surely $\left( R_{n}\right)$ forms a Følner sequence, and Flatto’s result in [25] to show that among the visited boxes, the majority of them are *q*-fully visited by the $\mu '$-walk. More precisely, by [25], we have that

$$\begin{aligned} \lim _{n\rightarrow \infty }\frac{\left| T_{n}^{q-1}\right| }{n/\log ^{2}n}=c_{q-1} \text{ a.s., } \end{aligned}$$

where $T_n^{p}$ is the number of points visited at least once and at most *p* times by the random walk up to time *n*. Note that the results in [25] is proven for simple random walk on ${\mathbb {Z}}^{2}$, but the proof applies to any centered random walk on ${\mathbb {Z}}^{2}$ with finite $(2+\epsilon )$-moment, see the Remark after [25, Theorem 3.1]. If a visited box $\alpha$ is not *q*-fully visited, then it must contain a point of $T_{n}^{q-1}$. Therefore the number of visited boxes that are not *q*-fully visited is bounded by $T_{n}^{q-1}$+$\left| \partial R_{n}\right|$.

In a box $\alpha$ that is *q*-fully visited by the $\mu '$-walk up to time *n*, consider the restriction of lamp configuration $\Phi _{n}$ to $\alpha$. Recall that $B_{n}^{\alpha }$ denotes the set of vertices in $\alpha$ without good update up to time *n*. As in Lemma 4.2, the configuration $\Phi _{n}|_{\alpha }$ can be written as $U_{\alpha }+\Delta _{n}^{\alpha }$, where the distribution of $U_{\alpha }$ is uniform on $\{0,1\}^{\alpha }$ and $\Psi _{n}$ is a random configuration with support on the set $B_{\alpha }$. It follows that to write the configuration $\Phi _{n}|_{\alpha }$ one can first write $U_{\alpha }$, then correct it by $\Delta _{n}^{\alpha }$ which is supported on $B_{\alpha }^{n}$, a subset of small size.

To prove the statement, we show that typically $|X_{n}|_{S}$ is approximated by the contribution from *q*-fully visited box $\sum _{\alpha \text{ is } q\text{-full }}\ell _{\mathrm{TS}}^{(G,S)}(U_{\alpha })$ with error bounded by $\epsilon n/\log n$.

**The upper bound for** $\left| X_{n}\right| _{S}$: we exhibit an *S*-path that connects the identity to $X_{n}$. Recall that we have divided ${\mathbb {Z}}^2$ into boxes of side length $c_n$. Consider all visited boxes and denote by ${\mathcal {P}}_n$ the union of the perimeters of the visited boxes. Because the step distribution $\mu$ allows long range jumps, the union of the perimeters of the visited boxes are not necessarily connected. We add paths to make the union of the perimeters connected. Note that it is sufficient to add connecting paths with total length bounded by the sum of random walk jumps longer than $c_{n}$. Indeed, we may enumerate the visited boxes according to the order they are visited as $\alpha _{1},\alpha _{2},...$: $\alpha _{i}$ is the first box visited by the ${\bar{\mu }}$-random walk after $\alpha _{1},...,\alpha _{i-1}$ that is different from the previous boxes. If the box $\alpha _{k}$ is not connected to $\alpha _{1}\cup ...\cup \alpha _{k-1}$, then we consider the random walk step that first enters $\alpha _{k}$ and take a shortest path connected the starting and ending point of this step. The union of such additional paths makes the union of the visited boxes connected and the length of the additional paths is bounded by

$$\begin{aligned} A_{n}=\sum _{i=1}^{n}|Y{}_{i}|_{S}{} \mathbf{1}_{\{|Y_{i}|_{S}\ge c_{n}\}}. \end{aligned}$$

Take first an *S*-path that starts at identity and covers the perimeters of each visited box, where connecting paths are added according to the long jumps of the random walk as described above. To connect *id* to $X_{n}$ by an *S*-path, along this path in each box $\alpha _{i}$, we take the TSP of the configuration $\Phi _{n}|_{\alpha _{i}}$ which starts and ends on the perimeter of $\alpha _{i}$. Then we can bound the length of the path by

4.1 $$\begin{aligned} |X_{n}|_{S}&\le \sum _{\alpha \ \text{ is } q\text{-full }}\left( \ell _{\mathrm{TS}}^{(G,S)}(U_{\alpha })+\ell _{\mathrm{TS}}^{(G,S)}(B_{n}^{\alpha })\right) \nonumber \\&\quad +C_0[c_{n}^{2}\left| \left\{ \alpha :\alpha \text{ is } \text{ visited } \text{ but } \text{ not } q\text{-full }\right\} \right| +|{\mathcal {P}}_{n}|]+A_{n}, \end{aligned}$$

where $C_0$ is a constant that only depends on the generating set *S*. Now we choose appropriate $c_{n}$ such that the main contribution to the quantity on the righthand side of (4.1) is from $\sum _{\alpha \ \text{ is } q\text{-full }}\ell _{\mathrm{TS}}^{(G,S)}(U_{\alpha })$. To this end, we need the expectation of $A_{n}$ to satisfy

4.2 $$\begin{aligned} {\mathbb {E}}[A_{n}]=n\sum _{|g|>c_{n}}|g|\mu (g)\ll n/\log n; \end{aligned}$$

and at the same time to be not too large so that the main contribution still comes from fully visited boxes. More precisely, we need $c_{n}$ to satisfy both the equation (4.2) above and

$$\begin{aligned} c_{n}^{2}\left( \left| R_{n}^{q-1}\right| +\left| \partial R_{n}\right| \right) \ll n/\log n. \end{aligned}$$

Since $\mu$ is assumed to have finite second moment, we have that $\delta _{r}=\sum _{|g|\ge r}|g|^{2}\mu (g)\rightarrow 0$ as $r\rightarrow \infty$. Thus we can choose $c_{n}$ to be

4.3 $$\begin{aligned} c_{n}:=\sqrt{\delta _{\log n}\log n}. \end{aligned}$$

**The lower bound for** $\left| X_{n}\right| _{S}$: given an *S*-path connecting *id* to $X_{n}$, we consider its restriction to each *q*-full box $\alpha$ and use the uncrossing Lemma 3.3 to show that the restriction can be modified to provide paths that write the configuration $U_{\alpha }$ in $\alpha$. The restriction of an *S*-path to $\alpha$ means the steps of the path with at least one of whose starting and ending points in $\alpha$. When the path is restricted to a box $\alpha$, we obtain a collection ${\mathcal {A}}_{\alpha }$ of paths, each with projection to ${\mathbb {Z}}^{2}$ starting and ending near the boundary of $\alpha$. More precisely, there is a constant $K>0$ which only depends on the generating set *S*, such that the starting and ending points of the paths in ${\mathcal {A}}_{\alpha }$ are within distance *K* to the boundary of $\alpha$. Reversing the directions of some paths if necessary, by concatenating paths we may assume that in the collection ${\mathcal {A}}_{\alpha }$, two distinct paths do not share common starting/ending points. In particular, the number of paths in the collection is bounded by $K'c_{n}$, where $K'$ is a constant only depending on *K*. For each path *P* with starting point *A* and ending point *B*, let $A'$ ($B'$ resp.) be the point on the boundary of $\alpha$ closest to *A* (*B* resp.). Replace *P* by the path $P'$ which is the concatenation of the shortest *S*-path from $\left( A',\mathbf{0}\right)$ to $\left( A,\mathbf{0}\right)$, the path *P* and the shortest path $\left( B,\tau _{P}\right)$ to $\left( B',\tau _{p}\right)$. Note that $|P'|\le |P|+2K$. Denote by ${\mathcal {A}}_{\alpha }'$ the collection of modified paths obtained from ${\mathcal {A}}_{\alpha }$. The purpose of making such modifications is to assure that paths in the collection ${\mathcal {A}}_{\alpha }'$ start and terminate on the boundary of $\alpha$. Applying the uncrossing Lemma 3.3 to the collection ${\mathcal {A}}_{\alpha }'$, we obtain a new collection ${\mathcal {B}}_{\alpha }$ of paths without essential crossings, such that ${\mathcal {B}}_{\alpha }$ produces the lamp configuration as ${\mathcal {A}}_{\alpha }$; and moreover the total length of paths in ${\mathcal {B}}_{\alpha }$ is bounded by

$$\begin{aligned} \sum _{Q\in {\mathcal {B}}_{\alpha }}|Q|\le \sum _{P'\in {\mathcal {A}}_{\alpha }'}|P'|+Cc_{n}\le \sum _{P\in {\mathcal {A}}_{\alpha }}|P|+C'c_{n}, \end{aligned}$$

where *C* and $C'$ are constants only depending on the generating set *S*. Reversing the direction of some paths in ${\mathcal {B}}_{\alpha }$ if necessary (this is allowed because the lamp group is ${\mathbb {Z}}/2{\mathbb {Z}}$, see Remark 3.2), we can connect the paths in ${\mathcal {B}}_{\alpha }$ along the perimeter of $\alpha$. This way we produce an *S*-path which produces the configuration $\Phi _{n}|_{\alpha }$, and the length of this path is bounded from above by $\sum _{P\in {\mathcal {A}}_{\alpha }}|P|+C''c_{n}$. To produce the configuration $U_{\alpha }$, we continue with a path which writes the difference $\Delta _{n}^{\alpha }$. Summing over the *q*-full boxes, we obtain the lower bound

4.4 $$\begin{aligned} |X_{n}|_{S}+\sum _{\alpha \ \text{ is } q\text{-full }}\left( C''c_{n}+\ell _{\mathrm{TS}}^{(G,S)}(B_{n}^{\alpha })\right) \ge \sum _{\alpha \ \text{ is } q\text{-full }}\ell _{\mathrm{TS}}^{(G,S)}(U_{\alpha }). \end{aligned}$$

**End of the proof**: with the bounds (4.1) and (4.4), the choice of $c_{n}$ as in (4.3), it suffices to show that a.s.,

$$\begin{aligned} \lim _{n\rightarrow \infty }\frac{1}{n/\log n}\sum _{\alpha \ \text{ is } q\text{-full }}\ell _{\mathrm{TS}}^{(G,S)}(U_{\alpha })&=c\\ \lim _{n\rightarrow \infty }\frac{1}{n/\log n}\sum _{\alpha \ \text{ is } q\text{-full }}\ell _{\mathrm{TS}}^{(G,S)}(B_{n}^{\alpha })&=0. \end{aligned}$$

By the law of large numbers of arrays of independent random variables to $\left\{ \ell _{\mathrm{TS}}^{(G,S)}(U_{\alpha })\right\}$ and $\left\{ \ell _{\mathrm{TS}}^{(G,S)}\left( B_{n}^{\alpha }\right) \right\}$, the first limit follows from Lemma 3.1 and the second limit follows from Lemmas 4.2 and 4.3. $\square$

### Remark 4.4

The only place where we use the $(2+\epsilon )$-moment condition in the proof is in Flatto’s result [25]. Recall that $T_n^{p}$ denotes the number of points visited at least once and at most *p* times by the random walk on ${\mathbb {Z}}^2$ up to time *n*. We have shown that if we have a centered non-degenerate probability measure $\mu$ of finite second moment on *G*, such that its projection to ${\mathbb {Z}}^2$ satisfies that for any $q\ge 2$,

$$\begin{aligned} \limsup _{n\rightarrow \infty }\frac{\left| T_{n}^{q-1}\right| }{n/\log ^{2}n}<\infty \text{ almost } \text{ surely, } \end{aligned}$$

then the statement of Theorem 1.1 would be true for $\mu$.

### Remark 4.5

For wreath product ${\mathbb {Z}}^{2}\wr F$ where *F* is an arbitrary non-trivial finite group, we say that a generating set *S* is complete, if for any $s=(z,f)\in S$, the element $(-z,\tau _{-z}f)$ is also in the generating set *S*. When $F={\mathbb {Z}}/2{\mathbb {Z}}$, the completeness of a generating set *S* is equivalent to symmetry of *S*. The proof of Theorem 1.1 extends to symmetric non-degenerate random walks of finite $(2+\epsilon )$-moment on ${\mathbb {Z}}^{2}\wr F$, when the length function $l_{S}$ is associated with a complete generating set *S*.

### Remark 4.6

**Fig. 7:**
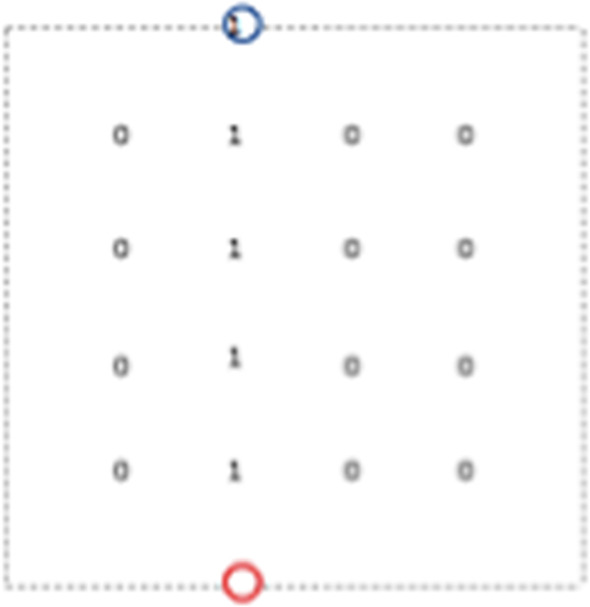
The picture illustrates in ${\mathbb {Z}}^2\wr {{\mathbb {Z}}/3{\mathbb {Z}}}$ a configuration on a square that the shortest length *S*-path traveling from bottom to top is much shorter than an *S*-path from top to bottom

When the generating set *S* of the wreath product ${\mathbb {Z}}^{2}\wr F$ is not complete, there are configurations where one cannot replace an *S*-path by an *S*-path of comparable length reversing the starting and ending points on ${\mathbb {Z}}^{2}$. For example, take $F={\mathbb {Z}}/3{\mathbb {Z}}$ and the generating set *S* which consists of $\left( e_{1},\mathbf{0}\right) ,\left( e_{2},\mathbf{0}\right)$, $\left( e_{2},\delta _{0}^{1}\right)$ and their inverses. In a box of side length *m*, consider the lamp configuration which is 1 on one vertical line and 0 everywhere else, see Fig. 7. Then for such a configuration, the optimal *S*-path whose projection to ${\mathbb {Z}}^{2}$ travels from bottom to top (red to blue in the picture) is of length *m*; while the optimal *S*-path which travels from bottom to top is of length at least 2*m*.

## Wreath products with infinite lamp groups

In this section we discuss some examples of wreath products with infinite lamp groups. Consider the wreath product $B\wr H=\left( \oplus _{x\in B}H\right) \rtimes B$, where both *B* and *H* are infinite. Let *P* be a generating set of *B* and *T* a generating set of *H*. We equip $B\wr H$ with the generating set $S=\left\{ \left( s,id\right) ,s\in P\right\} \cup \left\{ \left( id_{B},\delta _{id_{B}}^{t}\right) ,t\in T\right\}$, where $\delta _{id_{B}}^{t}:B\rightarrow H$ is the function that takes value *t* at $id_{B}$ and $id_{H}$ otherwise. Denote by $\left| \cdot \right| _{S}$ the word length on $B\wr H$ with respect to the generating set *S*.

Assume that *B* is ${\mathbb {Z}}$ or ${\mathbb {Z}}^{2}$ and *H* is a finitely generated infinite group. Take a symmetric nondegenerate probability measure $\nu$ of finite support on *B* and a symmetric nondegenerate probability measure $\eta$ on *H*. On the wreath product $B\wr H$, take the switch-walk-switch distribution $\mu =\eta *\nu *\eta .$ In addition we assume that the drift of the $\eta$-random walk on *H* with respect to word length $\left| \cdot \right| _{T}$ has finite second moment and satisfies

5.1 $$\begin{aligned} \lim _{t\rightarrow \infty }\frac{L_{\eta }(t)}{t^{\alpha }}=c \end{aligned}$$

for some constants $\alpha \in [1/2,1]$ and $c\in (0,\infty )$.

With respect to the generating set *S* we have for a group element $\left( z,f\right) \in B\wr H$,

5.2 $$\begin{aligned} \sum _{x\in \mathrm{supp}(f)}|f(x)|_{T}\le |(z,f)|_{S}\le \sum _{x\in \mathrm{supp}(f)}|f(x)|_{T}+2|\mathrm{supp}f|+|z|_{P}. \end{aligned}$$

Let $\left( X_{n}\right) _{n=0}^{\infty }$ be a random walk on $B\wr H$ with step distribution $\mu$ and write $X_{n}=({\bar{X}}_{n},\Phi _{n})$. Since the lamp group *H* is assumed to be infinite and the rate of escape of the $\eta$-random walk on *H* satisfies (5.2), the length estimate (5.2) shows that the main contribution to $\left| X_{n}\right| _{S}$ is from $\sum _{x}|\Phi _{n}(x)|_{T}$. Denote by $\left( Y_{t}^{x}\right) _{t=0}^{\infty }$ a collection of independent $\eta$-random walks, indexed by $x\in B.$ Let *l*(*n*, *x*) be the number of visits to $x\in B$ by the projected random walk ${\bar{X}}=({\bar{X}}_{n})_{n=0}^{\infty }$ on the base. Then $\left( \Phi _{n}(x)\right) _{x\in B}$ has the same distribution as $\left( Y_{l(n,x)}^{x}\right) _{x\in B}$. In particular, conditioned on the local times $\left( l(n,x)\right) _{x\in B}$, the random variables $\Phi _{n}(x)$, $x\in B$, are independent. Write $L_{\eta }(t)={\mathbb {E}}\left[ |Y_{t}^{x}|_{T}\right]$ and $V_{\eta }(t)=\mathrm{Var}\left( |Y_{t}^{x}|_{T}\right)$. Then by the law of large numbers for independent random variables, we have almost surely

5.3 $$\begin{aligned} \lim _{n\rightarrow \infty }\frac{\sum _{x\in R_{n}}\left| Y_{l(n,x)}^{x}\right| _{T}}{\sum _{x\in R_{n}}l(n,x)^{\alpha }}\rightarrow c, \end{aligned}$$

where *c* is the limit of $L_{\eta }(t)/t^{\alpha }$, as assumed in (5.1). The problem is reduced to the expression $\sum _{x\in R_{n}}l(n,x)^{\alpha }$ of local times on the base.

For example, when $H={\mathbb {Z}}$ and $\eta$ is a symmetric nondegenerate probability measure on ${\mathbb {Z}}$ with finite second moment, then the assumption (5.2) is satisfied with $\alpha =1/2$ and the variance satisfies $V_{\eta }(t)\simeq t$.

Now we consider an example with ${\mathbb {Z}}$ as a base group.

### Example 5.1

Let $B={\mathbb {Z}}$ and $H={\mathbb {Z}}$. Take the probability measure $\nu$ on *B* to be uniform on $\{\pm 1\}$ and take $\eta$ on *H* to be uniform on $\left\{ \pm 1\right\}$. On ${\mathbb {Z}}\wr {\mathbb {Z}},$ consider the random walk $\left( X_{n}\right) _{n=0}^{\infty }$ with the standard SWS step distribution $\mu =\eta *\nu *\eta$. Then by the invariance principle for local times of simple random walk on ${\mathbb {Z}}$ due to Perkins [46], we have that

$$\begin{aligned} \frac{1}{n^{3/4}}\sum _{x\in {\mathbb {Z}}}l(n,x)^{1/2}\Rightarrow \int _{-\infty }^{\infty }\left( L_{1}^{x}\right) ^{1/2}dx, \end{aligned}$$

where $L_{t}^{x}$ is the local time of the standard Brownian motion.

Next we return to the case of ${\mathbb {Z}}^{2}$ as a base group. Compared to Theorem 1.1, it is easier to establish the law of large numbers for random walks on the wreath product over ${\mathbb {Z}}^{2}$ with an infinite group as lamps. For a switch-walk-switch random walk on ${\mathbb {Z}}^{2}\wr {\mathbb {Z}}$, the problem is reduced to functionals of local times on ${\mathbb {Z}}^{2}$, where a result of Černý [13] can be applied.

### Example 5.2

Let $B={\mathbb {Z}}^{2}$ and $H={\mathbb {Z}}$. Let $\left( X_{n}\right) _{n=0}^{\infty }$ be a random walk on ${\mathbb {Z}}^{2}\wr {\mathbb {Z}}$ with switch-walk-switch step distribution $\eta *\nu *\eta$, where $\eta$ is uniform on $\{\delta ^{-1},\delta \}$ and $\nu$ is uniform on $\{s_{1}^{\pm 1},s_{2}^{\pm 1}\}$. Then there exists a constant $c\in (0,\infty )$

$$\begin{aligned} \lim _{n\rightarrow \infty }\frac{|X_{n}|_{S}}{n/\log ^{1/2}n}=c \text{ a.s. } \end{aligned}$$

### Proof

By Černý’s result [13], we know that there is a constant $C>0$ such that almost surely

5.4 $$\begin{aligned} \lim _{n\rightarrow \infty }\frac{\sum _{x\in R_{n}}l(n,x)^{1/2}}{n(\log n)^{-1/2}}=C. \end{aligned}$$

The claim in the example follows from combining (5.3) and (5.4), and the fact that the other two terms on the righthand side of (5.2) satisfy

$$\begin{aligned} \left| \mathrm{supp}\Phi _{n}\right|&\le |R_{n}| \text{ and } \lim _{n\rightarrow \infty }|R_{n}|/n(\log n)^{-1/2}=0\ \text{ a.s. },\\&\lim _{n\rightarrow \infty }|{\bar{X}}_{n}|/n(\log n)^{-1/2}=0 \text{ a.s. } \end{aligned}$$

$$\square $$

## Controlled Følner pairs and limiting behavior of $l_{S}(X_{n})/L_{\mu }(n)$

In general $l_{S}(X_{n})/L_{\mu }(n)$ does not necessarily converge in distribution. A strong opposite of concentration of $l_{S}(X_{n})$ around its mean is that $l_{S}(X_{n})/L_{\mu }(n)$ converges in distribution, and the limiting distribution admits a density which is supported on the whole ray $(0,\infty )$. In this section we consider the class of groups which admits controlled Følner pairs.

### Questions

Følner pairs were introduced in Coulhon et al. [15] to produce lower bounds on return probability. We recall the definition: a sequence $(F'_{n},F_{n})$ of pairs of finite subsets of *G* with $F'_{n}\subset F_{n}$ is called a sequence of *Følner pairs* adapted to an increasing function ${\mathcal {V}}(n)$ if there is a constant $C<\infty$ and such that for all *n*, we have

**(i)**: $$\#F_{n}\le C\#F'_{n}$$,
**(ii)**: $$d(F'_{n},G{\setminus } F_{n})\ge n$$,
**(iii)**: $$\#F_{n}\le {\mathcal {V}}(Cn)$$.

Følner pairs provide lower bound for return probability $\mu ^{(2n)}(e)$ for a symmetric probability measure $\mu$ of finite support on *G*, see [15]. The definition of controlled Følner pairs was introduced by Tessera in [50]. We say $(F'_{n},F_{n})$ with $F'_{n}\subset F_{n}$ is a sequence of *controlled Følner pairs* if they satisfy (i) and (ii) as above, and

**(iii’)**: $$F_{n}\subset B(e,Cn)$$.

Note that it follows from definition that controlled Følner pairs are adapted to the volume growth function of the group.

We ask the following question and in this section provide some evidence supporting a positive answer.

#### Question 6.1

Is it true that admitting controlled Følner pairs implies convergence of $l_{S}(X_{n})/L_{\mu }(n)$ in distribution and the limiting distribution admits a positive density on $(0,\infty )$?

In the context of the question, we mention the following asymptotic properties of finitely generated groups.

1. The Følner function is equivalent to the growth function $v_{S}(n)$.
2. The group admits a sequence of Følner pairs $\left( F_{n}',F_{n}\right)$ with volume of $F_{n}$ equivalent to the growth function $v_{S}(n)$.
3. The group admits controlled Følner pairs.
4. The return probability ${\mathbb {P}}(X_{2n}=e)$ of simple random walk to the origin is equivalent to the solution $\psi (n)$ of
  $$\begin{aligned} \int _{1}^{1/\psi (t)}\frac{1}{sv_{S}^{-1}(s)^{2}}ds=t. \end{aligned}$$
5. Simple random walk on the group has diffusive rate of escape, that is, $L_{\mu }(n)\simeq \sqrt{n}$.
6. Simple random walk on the group is cautious (we will recall the definition of cautiousness in Sect. 6.3).
7. The rate of escape is diffusive and the distribution of $|X_{n}|_{S}/L_{\mu }(n)$ converges in distribution to a limiting density whose support is $(0,\infty )$.

So far there is no known example that satisfies one of the conditions in the list but not all the others. All known examples satisfying these conditions are elementary amenable, in particular, there is no known example of group of intermediate growth with properties in the list. One can also ask when the rate of escape is diffusive, whether the entropy of the random walk is equivalent to $\log v_{S}\left( \sqrt{n}\right)$. Another question one can ask in the setting of condition 7 is whether $|X_{n}|_{S}/L_{\mu }(n)$ exhibits Gaussian tail decay, that is, there exists a constant $c>0$ such that

$$\begin{aligned} {\mathbb {P}}\left( |X_{n}|_{S}/L_{\mu }(n)\ge x\right) \le e^{-cx^{2}} \text{ for } x>1. \end{aligned}$$

We remark that properties in the list 1-6 describe some asymptotic properties of the group that are minimal possible. Recall that by [35], the rate of escape of simple random walk on an infinite group has a diffusive lower bound. Property 5 in the list describes that the rate of escape is minimal possible in the sense that it is equivalent to the universal lower bound. By the Coulhon-Saloff-Coste inequality, the Følner function is at least the volume growth. For example, on a group of exponential volume growth, the Coulhon-Saloff-Coste inequality implies that the Følner function is at least exponential and the return probability satisfies ${\mathbb {P}}(X_{2n}=e)\preceq e^{-n^{1/3}}$. Property 1 and 4 describe that the Følner function (return probability of simple random walk resp.) is minimal (maximal resp.) possible given the volume growth. Properties 2 and 3 are strengthenings of Property 1, concerning not only the cardinalities of Følner sets, but also the shape and geometry of them.

One class of groups where the positive answer to Question 6.1 is known consists of groups of polynomial growth. Note that groups of polynomial growth admit controlled Følner pairs. As already mentioned in the Introduction, on groups of polynomial growth, one can apply the local limit theorem in Alexopoulos [1]. More precisely, let *G* be a group of polynomial growth and $\mu$ be a symmetric probability on *G* with finite generating support. Then [1, Corollary 1.19] states that for any $\epsilon \in (0,1)$, there is a constant $C>0$ such that

$$\begin{aligned} \left| \mu ^{(n)}(x^{-1}y)-p_{n}^{H_{\mu }}\left( x,y\right) \right| \le Cn^{-\frac{1}{2}(D+\epsilon )}\exp \left( -\frac{l_{S}(x^{-1}y)^{2}}{Cn}\right) , \end{aligned}$$

where $H_{\mu }$ is the limiting Laplacian on a nilpotent Lie group *N* which is referred to as the stratified Lie group associated to *G* in [1]. The stratified nilpotent group *N* admits a dilation $\delta :N\rightarrow N$ and the transition probabilities of the limiting process is scale invariant with respect to the dilation: $p_{st}^{H_{\mu }}\left( \delta _{\sqrt{s}}x,\delta _{\sqrt{s}}y\right) =p_{t}^{H_{\mu }}(x,y)$. The local limit theorem implies that $l_{S}(X_{n})/\sqrt{n}$ converges in distribution and the limiting distribution admits a positive density on ${\mathbb {R}}_{+}$.

### The example of ${\mathbb {Z}}\wr F$

In this subsection we explain the case of the lamplighter ${\mathbb {Z}}\wr F$ over ${\mathbb {Z}}$ with the lamp group *F* a finite nontrivial group. The group ${\mathbb {Z}}\wr F$ is an example of a group of exponential growth that admits controlled Følner pairs. We show that for simple random walk on ${\mathbb {Z}}\wr F$, the limiting distribution of $l_{S}(X_{n})/L_{\mu }(n)$ satisfies the claim of Question 6.1. Let $\left( B_{t}\right) _{t\ge 0}$ be the standard Brownian motion and denote by ${\mathcal {R}}_{t}$ the size of the range of the standard Brownian motion $\left( B_{t}\right) _{t\ge 0}$ at time *t*.

#### Proposition 6.2

Let $\mu$ be a non-degenerate symmetric measure of finite second moment on ${\mathbb {Z}}\wr F$, *F* is a finite group, $F\ne \left\{ id\right\}$. Let *S* be a finite symmetric generating set of ${\mathbb {Z}}\wr F$. Then there exists constants $c_{1},c_{2}>0$ such that $l_{S}\left( X_{n}\right) /\sigma \sqrt{n}$ converges in distribution to $c_{1}\left( {\mathcal {R}}_{1}-|B_{1}|\right) +c_{2}|B_{1}|$, where $\sigma$ is the variance of ${\bar{X}}_{1}$.

#### Proof

We first explain a particular case which is easier. Consider the SWS generating set $S_{0}$ which consists of elements of the form $\left( e_{1},\delta _{0}^{\gamma }+\delta _{1}^{\gamma '}\right)$, where $\gamma ,\gamma '\in F$, and their inverses. Take the random walk step distribution $\mu _{0}$ to be uniform on $S_{0}$. Recall that for an element $(x,f)\in ({\mathbb {Z}}/2{\mathbb {Z}})\wr {\mathbb {Z}}$, for the SWS generating set $S_{0}$, we have

$$\begin{aligned} l_{S_{0}}\left( \left( x,f\right) \right) =2\max \left\{ \mathrm{supp}f\right\} -2\min \left\{ \mathrm{supp}f\right\} -|x|, \end{aligned}$$

if $x\in \left[ \min \left\{ \mathrm{supp}f\right\} ,\mathrm{max}\left\{ \mathrm{supp}f\right\} \right]$. Denote by $R_{n}$ the range of the projected $\mu _{0}$-random walk on ${\mathbb {Z}}$ at time *n*. Then there is a constant $C>0$, almost surely for all *n* sufficiently large,

6.1 $$\begin{aligned} \left| l_{S}\left( X_{n}\right) -\left( 2|R_{n}|-|{\bar{X}}_{n}|\right) \right| \le C\log n. \end{aligned}$$

In [29], a central limit theorem for $|R_{n}|$ is shown: $|R_{n}|/\sqrt{n}$ converges to ${\mathcal {R}}_{1}$ in distribution. This follows from Donsker’s invariance principle, and similarly one can show that $\left( 2|R_{n}|-|{\bar{X}}_{n}|\right) /\sqrt{n}$ converges to $2{\mathcal {R}}_{1}-|B_{1}|$ in distribution. Denote by $f_{\infty }$ the density of the random variable $2{\mathcal {R}}_{1}-|B_{1}|$. Thus by (6.1), we have that the limiting density of $l(X_{n})/\sqrt{n}$ is $f_{\infty }$.

Next we consider the general case. We argue in a way similar to the proof of Theorem 1.1, except that in the one-dimensional case the uncrossing lemma is not needed. Note the following consequence of sub-additivity.

#### Lemma 6.3

Let $\mathcal {\Lambda }_{n}$ be a configuration uniform on $\oplus _{z\in \left\{ 1,\ldots ,n\right\} }(F)_{z}$. Denote by $\mathrm{TSP}_{S}\left( \Lambda _{n};x,y\right)$ the shortest *S*-path that writes $\Lambda _{n}$ whose projection in ${\mathbb {Z}}$ starts at *x* and ends at *y*. Then there are constants $c_{1},c_{2}>0$ such that almost surely

$$\begin{aligned} \lim _{n\rightarrow \infty }\frac{1}{n}\mathrm{TSP}_{S}\left( \Lambda _{n};1,1\right)&=c_{1},\\ \lim _{n\rightarrow \infty }\frac{1}{n}\mathrm{TSP}_{S}\left( \Lambda _{n};1,n\right)&=c_{2}. \end{aligned}$$

#### Proof of Lemma 6.3

Let $\Lambda$ be the i.i.d. uniform configuration on $F^{{\mathbb {Z}}}.$ Given two integers $x\le y$, denote by $\Lambda _{[x,y]}$ the restriction of $\Lambda$ to the interval $\{x,\ldots ,y\}$. Then there is a constant *C* which depends only on *S* such that for any $x\le y\le z$, we have

6.2 $$\begin{aligned} \mathrm{TSP}_{S}\left( \Lambda _{[x,z]};x,x\right) \le \mathrm{TSP}_{S}\left( \Lambda _{[x,y]};x,x\right) +\mathrm{TSP}_{S}\left( \Lambda _{[y,z]};y,y\right) +C. \end{aligned}$$

Indeed if we have paths $P_{1}$ of optimal length starting and ending at *x* producing $\Lambda _{[x,y]}$, and $P_{2}$ of optimal length starting and ending at y producing $\Lambda _{[y,z]}$, then we may obtain the following path which writes $\Lambda _{[x,z]}$. Follow $P_{1}$, which starts at *x*, until the first time it exits the interval $\left[ x,y\right]$, call this segment of $P_{1}$ as $P_{1}'$. Denote by $y'$ the end point of $P_{1}'$, it is within bounded distance $C_{0}$ to *y*. Then to write $\Lambda _{[x,z]}$, we first follow $P_{1}'$, then move from $y'$ to *y* without changing the configuration, then follow $P_{2}$, move back from *y* to $y'$, and finally follow the rest of $P_{1}$. The inequality (6.2) follows.

By concatenating *S*-paths, we have that

6.3 $$\begin{aligned} \mathrm{TSP}_{S}\left( \Lambda _{[x,z]};x,z\right) \le \mathrm{TSP}_{S}\left( \Lambda _{[x,y]};x,y\right) +\mathrm{TSP}_{S}\left( \Lambda _{[y,z]};y,z\right) +C. \end{aligned}$$

Given (6.2) and (6.3), the statement follows from Kingman’s subadditive ergodic theorem. $\square$

Now we return to the proof of Proposition 6.2. We show that there exist constants $c_{1},c_{2}>0$ (these will be the constants in Lemma 6.3) such that almost surely

6.4 $$\begin{aligned} \lim _{n\rightarrow \infty }\frac{l_{S}\left( X_{n}\right) }{c_{1}(R_{n}-|{\bar{X}}_{n}|)+c_{2}|{\bar{X}}_{n}|}=1, \end{aligned}$$

where ${\bar{X}}_{n}$ is the projection of $X_{n}$ to ${\mathbb {Z}}$ and $\left| {\bar{X}}_{n}\right|$ is its absolute value.

The proof of (6.4) is similar to that of Theorem 1.1, except that the 1-dimensional case is easier and does not require the uncrossing lemma (while the uncrossing lemma is essential in the 2-dimensional case in Theorem 1.1). Given any constant $\epsilon >0$, at time *n* choose $c_{n}$ and divide ${\mathbb {Z}}$ into intervals of length $c_{n}$. Note that by [16, Theorem 21] (see also the remark after it), we have that for any $\delta >0$, almost surely

$$\begin{aligned} \frac{\left| \partial R_{n}\right| }{|R_{n}|}=o\left( n^{-\frac{1}{2}+\delta }\right) . \end{aligned}$$

**Fig. 8:**
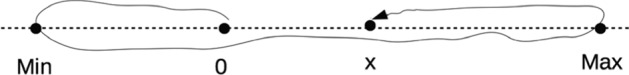
Given a prescribed configuration on $[\min ,\max ]$ and end point $x_{n}$, on the subintervals [*min*, 0] and [*x*, *max*] we have TSP problem of the first kind; on the interval [0, *x*] we have TSP problem of the second kind. This results in the two constants $c_{1},c_{2}$

We proceed in the same way as in the proof of Theorem 1.1. In the upper bound direction, to obtain an *S*-path we concatenate paths lying inside each interval and connect disjoint intervals to the rest of the intervals if necessary. In this way we obtain an upper bound for $l_{S}(X_{n})$ analogous to (4.1). In the lower bound direction, we start with an optimal *S*-path connecting id to $X_{n}$, then up to bounded error, in each interval it induces a TSP path either of the first or second kind, see Fig. 8. In this way we obtain a lower bound analogous to (4.4). The convergence to the limit as in (6.3) is proved in the same way as in Theorem 1.1.

Similar to the SWS random walk case, as a consequence of Donsker’s invariance principle, the expression $c_{1}(R_{n}-|{\bar{X}}_{n}|)+c_{2}|{\bar{X}}_{n}|$ normalized by $\sigma \sqrt{n}$, converges in distribution to $c_{1}\left( {\mathcal {R}}_{1}-|B_{1}|\right) +c_{2}|B_{1}|$. Combined with (6.4), we conclude that $l_{S}(X_{n})/\sigma \sqrt{n}$ converges in distribution to $c_{1}\left( {\mathcal {R}}_{1}-|B_{1}|\right) +c_{2}|B_{1}|$. $\square$

### A partial result in the general case of groups admitting controlled Følner pairs

In this subsection we discuss some general properties of groups admitting controlled Følner pairs.

If a group *G* admits a sequence of controlled Følner pairs, then simple random walk on *G* is diffusive, that is, $L_{\mu }(n)\simeq \sqrt{n}$, see [45, Theorem 1.4] (the statement is formulated in terms of $\ell ^{2}$-isoperimetric profile, by [15] controlled Følner pairs imply that the $\ell ^{2}$-isoperimetric profile in balls satisfy $\lambda \left( B(id,r)\right) \lesssim r^{-2}$). As introduced in [22], the random walk $(X_{n})_{n=0}^{\infty }$ is *cautious* if for any constant $c>0$, there exists a constant $\delta =\delta (c)>0$, such that ${\mathbb {P}}(l_{S}(X_{n})\le c\sqrt{n})\ge \delta (c)$. In particular, if the random walk $(X_{n})_{n=0}^{\infty }$ is *cautious*, then $l_{S}(X_{n})/L_{\mu }(n)$ does not converge in probability. Admitting a sequence of controlled Følner pairs implies that simple random walks are cautious, see [23, Lemma 4.5].

The following lemma shows that in the context of Question 6.1, if the limiting distribution exists, then it must be supported on the whole ray $\left( 0,\infty \right)$.

#### Lemma 6.4

Let *G* be a group where the $\mu$-random walk on *G* is cautious. Suppose in addition that $l_{S}(X_{n})/\sqrt{n}$ converges in distribution to a limiting distribution $\nu$. Then for any $(a,b)\subseteq [0,\infty )$, we have $\nu \left( \left( a,b\right) \right) >0$.

#### Proof

The cautiousness condition implies that for any $\varepsilon >0$, $\nu ((0,\varepsilon ))>0$. By [22], since the $\mu$-random walk $\left( X_{n}\right) _{n=0}^{\infty }$ on *G* is cautious, *G* admits a virtual homomorphism onto ${\mathbb {Z}}$. Denote by $G_{1}$ a finite index subgroup of *G* with a homomorphism $\pi :G_{1}\rightarrow {\mathbb {Z}}$ that is onto. Consider the induced random walk on $G_{1}$: let $\tau _{0}=0$, $\tau _{k}=\min \{n>\tau _{k-1}:X_{n}\in G_{1}\}$, and $Y_{k}=X_{\tau _{k}}$. By the law of large numbers, we have that $\tau _{k}/k\rightarrow {\mathbb {E}}[\tau _{1}]$ almost surely when $k\rightarrow \infty$. By the central limit theorem on ${\mathbb {Z}}$, we have that $\pi \left( X_{\tau _{k}}\right) /\sqrt{n}$ converges to normal distribution $N(0,\sigma ^{2})$ in distribution. Note that there is a constant *c*, which only depends on $G_{1}$ and the generating set *S*, such that $\left| X_{\tau _{k}}\right| _{S}\ge c\left| \pi \left( X_{\tau _{k}}\right) \right|$. Therefore for any $x>0$, we have

$$\begin{aligned} \lim _{n\rightarrow \infty }{\mathbb {P}}\left( \frac{\left| X_{n}\right| _{S}}{\sqrt{n}}\ge x\right)&=\lim _{k\rightarrow \infty }{\mathbb {P}}\left( \frac{\left| X_{\tau _{k}}\right| _{S}}{\sqrt{k{\mathbb {E}}\left[ \tau _{1}\right] }}\ge x\right) \\&\ge \lim _{n\rightarrow \infty }{\mathbb {P}}\left( \frac{c\left| \pi \left( X_{\tau _{k}}\right) \right| }{\sqrt{k{\mathbb {E}}\left[ \tau _{1}\right] }}\ge x\right) \\&=2\Phi \left( \frac{{\mathbb {E}}\left[ \tau _{1}\right] ^{1/2}}{c\sigma }x\right) , \end{aligned}$$

where $\Phi (x)={\mathbb {P}}(Z>x)$, *Z* the standard normal random variable. In particular, it follows that for any $x>0$, $\nu \left( \left( x,\infty \right) \right) >0$.

Given $0<a<b<\infty$, choose an interval (*A*, *B*) such that $A>b$, $|B-A|<|b-a|/2$ and $\nu \left( \left( A,B\right) \right) >0$. Such an interval (*A*, *B*) exists because $v((x,\infty ))>0$ for all $x>0$. Next, choose a constant $c\in (0,1)$ and a sufficiently small $\varepsilon >0$ such that

$$\begin{aligned} \left( A\sqrt{c}-\varepsilon ,B\sqrt{c}+\varepsilon \right) \subseteq \left( a,b\right) . \end{aligned}$$

For example, one can choose *c* to satisfy $A\sqrt{c}=a+\frac{1}{3}(b-a)$ and take $0<\varepsilon <\frac{1}{3}(b-a)$. By the triangle inequality, we have for $k=cn$,

$$\begin{aligned} {\mathbb {P}}\left( \left| X_{n}\right| {}_{S}\in \left( a\sqrt{n},b\sqrt{n}\right) \right) \ge {\mathbb {P}}\left( \left| X_{k}\right| _{S}\in \left( A\sqrt{k},B\sqrt{k}\right) \right) \cdot {\mathbb {P}}\left( \left| X_{n-k}\right| _{S}\le \varepsilon \sqrt{n-k}\right) . \end{aligned}$$

Then in the limit we have

$$\begin{aligned} v\left( \left( a,b\right) \right) \ge \nu ((A,B))\nu ((0,\epsilon ))>0. \end{aligned}$$

$$\square $$

#### Remark 6.5

As explained before the statement of Lemma 6.4, admitting controlled Følner pairs implies that simple random walk on *G* is cautious. Thus in the context of Question 6.1, if the limiting distribution exists and admits a density, then the support of the density must be the whole ray $(0,\infty )$.

### Dependence of the limiting distribution on subsequences

For groups which admit controlled Følner pairs only along a subsequence, the situation is more complicated. In this subsection we explain an example of a group *G* such that for a simple random walk $\mu$ on *G*, along two difference subsequences, the limiting distributions of $l_{S}(X_{n})/L_{\mu }(n)$ are different. Denote by $f_{\infty }$ the density function of $c_{1}\left( {\mathcal {R}}_{1}-|B_{1}|\right) +c_{2}|B_{1}|$ as in Proposition 6.2.

#### Proposition 6.6

There exists a finitely generated group *G*, which is locally-nilpotent-by-${\mathbb {Z}}$, such that along one subsequence $(n_{i})$, $l_{S}(X_{n_{i}})/L_{\mu }(n_{i})$ converges in distribution to the limiting density $f_{\infty }$; and along another subsequence $(m_{i}),$ $l_{S}(X_{m_{i}})/L_{\mu }(m_{i})$ converges in probability to constant 1.

#### Proof

Consider the locally-nilpotent-by-${\mathbb {Z}}$ groups which are constructed in [27]. We recall the construction: take a prime number *p* and denote by $\mathbf{M}$ the free product $*_{i\in {\mathbb {Z}}}\left\langle a_{i}\right\rangle$, where $a_{i}$ satisfies $a_{i}^{p}=1$, $i\in {\mathbb {Z}}$. Take a non-decreasing sequence $\mathbf{c}=\left( c_{i}\right) _{i=1}^{\infty }$ of positive integers and denote by $A(p,{\mathbf {c}})$ the quotient group of ${\mathbf {M}},$ subject to the relations that for each $n\in {\mathbb {N}}$,

$$\begin{aligned} \left[ \ldots \left[ a_{i_{0}},a_{i_{1}}\right] ,\ldots ,a_{i_{c_{n}}}\right] =1,\ \text{ where } \max _{j,k}\left| i_{j}-i_{k}\right| \le n. \end{aligned}$$

Denote by $G(p,\mathbf{c})$ the semi-product $A(p,\mathbf{c})\rtimes \left\langle t\right\rangle$, where *t* acts by the automorphism $a_{i}\rightarrow a_{i+1}$, $i\in {\mathbb {Z}}$, on $A(p,\mathbf{c})$.

As considered in [23], one can impose additional relators in $G(p,\mathbf{c})$ such that the group is close to the lamplighter ${\mathbb {Z}}\wr ({\mathbb {Z}}/p{\mathbb {Z}})$ along another subsequence. Let $G_{0}=({\mathbb {Z}}/p{\mathbb {Z}})\wr {\mathbb {Z}}$ and ${\mathbf {M}}=*_{i\in {\mathbb {Z}}}\left\langle b_{i}\right\rangle$. We define a sequence of quotients of $G(p,\mathbf{c})$ recursively as follows, parametrized by the sequence $(\ell _{i},c_{i},k_{i})_{i\in {\mathbb {N}}}$.

After we have defined $G_{i}$, given the parameter $\ell _{i+1}$, take the nilpotent subgroup in $G_{i}$ generated by $b_{0},\ldots ,b_{2^{\ell _{i+1}}-1}$,

$$\begin{aligned} N_{i+1}=\left\langle b_{0},\ldots ,b_{2^{\ell _{i+1}}-1}\right\rangle . \end{aligned}$$

Consider the quotient group ${\hat{M}}_{i+1}$ of *M* defined by imposing the relations that for any *j*, the subgroup generated by $\left\langle b_{j},\ldots ,b_{j+2^{\ell _{i+1}}-1}\right\rangle$ is isomorphic to $N_{i+1}$ (isomorphism given by shifting indices by *j*). Let ${\hat{\Gamma }}_{i+1}={\hat{M}}_{i+1}\rtimes {\mathbb {Z}}$ be the cyclic extension of ${\hat{M}}_{i+1}$. Then ${\hat{\Gamma }}_{i+1}$ splits as an HNN-extension of the finite nilpotent group $N_{i+1}$, therefore ${\hat{\Gamma }}_{i+1}$ is virtually free.

Given the parameters $c_{i+1},k_{i+1}\in {\mathbb {N}}$, consider the quotient group $G_{i+1}={\bar{\Gamma }}_{i+1}(c_{i+1},k_{i+1})$ of ${\hat{\Gamma }}_{i+1}$ subject to additional relations $(*)$

$$\begin{aligned} \left[ \left[ \left[ b_{j_{1}},b_{j_{2}}\right] ,b_{j_{3}}\right] ,\ldots ,b_{j_{m}}\right]&=0 \text{ for } \text{ any } m\ge c_{i+1},\\ b_{j}&=b_{j+2^{k_{i+1}}} \text{ for } \text{ all } j\in {\mathbb {Z}}. \end{aligned}$$

By construction, if we choose $\ell _{i+1}\gg \max \left\{ \ell _{i},k_{i},c_{i}\right\}$ and $k_{i+1},c_{i+1}\gg \ell _{i+1}$ to be large enough parameters, then we have that $G_{i+1}$ and $G_{i}$ coincide on the ball of radius $2^{\ell _{i+1}}$ around the identity element, and moreover, $G_{i+1}$ and ${\hat{\Gamma }}_{i+1}$ coincide on the ball of radius $2^{k_{i+1}}$.

On the group ${\mathbf {M}}\rtimes {\mathbb {Z}}$, denote by $\mu$ the uniform measure on $\left\{ t^{\pm 1},b_{0}^{\pm 1}\right\}$. In what follows, on a quotient group of ${\mathbf {M}}\rtimes {\mathbb {Z}}$, we consider the random walk with step distribution the projection of $\mu$.

We now specify the choice of parameters $(\ell _{i},k_{i},c_{i})$. Fix a sequence of positive numbers $\left( \epsilon _{i}\right) _{i=1}^{\infty }$ such that $\epsilon _{i}$ converges to 0 when $i\rightarrow \infty$. Suppose $G_{i}$ is defined and we choose $\ell _{i+1},k_{i+1}$ and $c_{i+1}$. By its definition $G_{i}$ is a finite extension of $({\mathbb {Z}}/p{\mathbb {Z}})\wr {\mathbb {Z}}$. More precisely, let $H_{i}$ be the subgroup of $G_{i}$ generated by $b_{0},\ldots ,b_{2^{k_{i}}-1}$. Note that $H_{i}$ is a finite nilpotent group. Then because of the relations $(*)$ in ${\bar{\Gamma }}_{i}$, $G_{i}$ fits into the exact sequence

$$\begin{aligned} 1\rightarrow [H_{i},H_{i}]\rightarrow G_{i}\rightarrow ({\mathbb {Z}}/p{\mathbb {Z}})\wr {\mathbb {Z}}\rightarrow 1. \end{aligned}$$

More precisely, one can recursively choose intervals $I_{k},J_{k}$ in ${\mathbb {N}}$ with $\max I_{k}<\min J_{k}$ and groups $H_{k},G_{k}$ such that

(i) $H_{k}$ is virtually free (and it only depends on $G_{1},H_{1},\ldots ,G_{k-1},H_{k-1},G_{k}$) and for an integer $r\in J_{k}$, the ball of radius *r* in $H_{k}$ around *id* coincides with the ball of the same radius around *id* in $G_{k}.$

(ii) for an integer $r\in I_{k}$, the ball of radius *r* in $G_{k}$ is the same as the ball of same radius in $H_{k-1}$; and $G_{k}$ is a finite extension of ${\mathbb {Z}}\wr ({\mathbb {Z}}/2{\mathbb {Z}})$ where the extension only depends on choices up to index $k-1$.

Let *G* be the direct limit of the sequence of groups $\{G_{k}\}.$ In the construction one can take the intervals $I_{k},J_{k}$ to be arbitrarily long. By the subadditive theorem, on the virtually free group $H_{k-1}$, we have $|X_{n}^{(k-1)}|_{S}/L_{(k-1)}(n)\rightarrow 1$ almost surely as $n\rightarrow \infty$. Thus we can choose $I_{k}$ to be long enough such that almost surely for all $n\ge m(I_{k})$, $\left| |X_{n}^{(k-1)}|_{S}/L_{(k-1)}(n)-1\right| <\epsilon _{k}$. To choose $J_{k}$, we make sure that it is sufficiently long such that diameter of kernel of $G_{k}\rightarrow {\mathbb {Z}}\wr ({\mathbb {Z}}/2{\mathbb {Z}})$ is less than $\epsilon _{k}m(J_{k})$.

It follows that if the intervals $I_{k},J_{k}$ are sufficiently long, in the direct limit *G*, along one subsequence of time, the random walk sees the virtually free group; and along another subsequence of time it sees the wreath product $\mathbb {Z\wr {\mathbb {Z}}}/2{\mathbb {Z}}$. $\square$

#### Remark 6.7

As mentioned in the proof, the groups of the form $G(p,\mathbf{c})$ are first considered in [27]. In terminology introduced later, with appropriate choice of ${\mathbf {c}}$, the group $G(p,\mathbf{c})$ is lacunary hyperbolic, see [42, Section 3.5]. Roughly speaking, being lacunary hyperbolic means that the group looks like hyperbolic groups along a subsequence of scales.
